# Nicotine-induced *CHRNA5* activation modulates *CES1* expression, impacting head and neck squamous cell carcinoma recurrence and metastasis via MEK/ERK pathway

**DOI:** 10.1038/s41419-024-07178-4

**Published:** 2024-10-29

**Authors:** Chen Feng, Wei Mao, Chenyang Yuan, Pin Dong, Yuying Liu

**Affiliations:** 1 Department of Otolaryngology, Head and Neck Surgery, Shanghai General Hospital, Shanghai Jiao Tong University School of Medicine, Shanghai, China; 2 Department of Otolaryngology, Head and Neck Surgery, Qilu Hospital, Shandong University Cheeloo College of Medicine, NHC Key Laboratory of Otorhinolaryngology (Shandong University), Jinan, China; 3 Department of Otorhinolaryngology, Head and Neck surgery, The First Hospital affiliated to Harbin Medical University, Harbin, Heilongjiang China

**Keywords:** Head and neck cancer, Diagnostic markers

## Abstract

The mucosal epithelium of the head and neck region (including the oral cavity, nasal cavity, pharynx, nasopharynx, and larynx) is the primary site exposed to tobacco smoke, and its presence of nicotinic acetylcholine receptors (nAChRs) has been observed in the mucosal epithelial cells of this area. It remains unclear whether HNSC cells can migrate and invade through nAChR signaling. A model of HNSC cells exposed to nicotine is established. Cell proliferation following nicotine exposure is assessed using the CCK-8 assay, while migration and invasion are evaluated through wound healing and Transwell assays. The effects of *CHRNA5* knockdown and overexpression are also investigated. Immunofluorescence staining is used to analyze *CHRNA5* expression and localization, and clonogenic assays are performed to measure colony proliferation after *CHRNA5* knockdown and overexpression. The interaction between CHRNA5 and CES1 is examined using molecular docking, co-immunoprecipitation, and immunofluorescence. Differentially expressed genes are subjected to pathway enrichment analysis, and MEK/ERK protein expression and phosphorylation are validated via western blot. Tumor formation assays are performed in nude mice using sh-*CHRNA5* Cal27 cells, followed by western blot and immunohistochemical staining. Additionally, laryngeal and hypopharyngeal cancer tissues are analyzed through immunohistochemistry. Nicotine significantly enhanced the proliferation, migration, and invasion capabilities of head and neck tumor cells, including Cal27, Fadu, HN6, and Tu686 cells, through the expression of *CHRNA5*. Knockdown of *CHRNA5* can reduce cell migration, invasion, and proliferation, whereas nicotine exposure can reverse this trend. Additionally, the mRNA and protein expression of CES1 decreases with the knockdown of *CHRNA5*, indicating a regulatory relationship between the two. Transcriptomics revealed that the knockdown of *CHRNA5* is associated with the MEK/ERK signaling pathway. Further cellular- and tissue-level evidence confirmed that the levels of p-MEK/MEK, p-ERK/ERK, and CES1 decreased following knockdown of *CHRNA5*, a trend that nicotine can reverse. Nicotine promotes the proliferation, migration, and invasion of HNSC by upregulating *CHRNA5* expression. Knockdown of *CHRNA5* reduces these effects, which can be reversed by nicotine. Nicotine exposure activates CHRNA5, regulating CES1 expression via the MEK/ERK pathway, contributing to the recurrence and metastasis of head and neck squamous carcinoma.

## Introduction

Head and neck tumors encompass a range of malignant neoplasms that can occur within various structures of the upper respiratory and digestive tracts and represent the seventh most common type of cancer globally [1, 2]. Head and neck squamous cell carcinoma (HNSC) is the most prevalent type among head and neck tumors, accounting for over 90% of cases [1, 2], with a mortality rate of 40–50% [3]. The early detection of HNSC remains challenging. As the tumor grows, it locally infiltrates and metastasizes to regional lymph nodes, resulting in over 60% of patients being diagnosed at advanced stages (stage III or IV) [4]. Previous research has suggested the possibility of multiple potential oncogenes in HNSC, involving aspects such as tumor metastasis and immune function regulation, with specific applications still pending further study [1]. While programmed death 1(PD-1) immune checkpoint inhibitors have been pivotal in treating HNSC and other cancers, the targeted molecular therapies have not brought significant benefits to patients [5, 6]. Consequently, there is an urgent need to utilize genetic and genomic approaches to discover new diagnostic and prognostic biomarkers for HNSC.

Since the 1950s, the link between smoking and HNSC has been widely recognized, with rising smoking rates in developing countries leading to an increased likelihood of smoking-induced tumors [1]. Emerging evidence indicates a correlation between smoking and an increased incidence of lung cancer, breast cancer, pancreatic cancer, esophageal cancer, and head and neck tumors [7–10]. Exposure of cells to nicotine alters the expression of various genes associated with cell migration and angiogenesis, accelerates the formation of atherosclerotic plaques and tumor development, and potentially plays a significant role in pathological angiogenesis [11–13]. Carcinogens derived from tobacco can directly affect the genome to cause the formation of DNA adducts and can also bind to nicotinic acetylcholine receptors (nAChRs) on the surface membrane of non-neuronal cells, activating growth-related intracellular signaling pathways, thereby promoting tumor initiation or progression [13, 14]. To date, 16 types of nAChRs have been identified in humans and are distributed in muscle and neural tissues. These nAChR subunits include α1-α7, α9, α10, β1-β4, γ, δ, and ε [15], corresponding to *CHRNA1-7, CHRNA9, CHRNA10, CHRNB1-4, CHRND, CHRNE*, and *CHRNG*. Scherl [16] analyzed 34 cases of squamous cell carcinoma of the hypopharynx, oropharynx, and larynx and 38 corresponding normal mucosa samples and found that *CHRNA1, CHRNA3, CHRNA5*, and *CHRNA7* were present in both the normal mucosa of the upper respiratory and digestive tract and in head and neck squamous carcinomas, with mRNA expression levels of *CHRNA5* and *CHRNA7* being higher than those of *CHRNA1* and *CHRNA3*. The relationship between the expression of various nAChR subunits and clinicopathological characteristics revealed that the expression of *CHRNA1* and *CHRNA5* increases with tumor progression [16]. Differences in nAChR expression between tumor surface cancer cells and normal mucosa suggest a potential role for nAChR in promoting tumor proliferation [17].

Nicotine, as an addictive substance, acts not only in the central nervous system but also in other cell types. The interaction between nicotine and nAChR activates the Mitogen-activated protein kinases (MAPK) pathway, promoting cell growth, angiogenesis, and related cellular activities [18–20]. The MAPK pathway plays a crucial role in the proliferation and survival of tumor cells, consisting of RAS (rat sarcoma virus (RAS), rapidly accelerated fibrosarcoma (RAF), mitogen-activated protein kinase kinase (MEK), and extracellular signal-regulated kinases (ERK), which transmit proliferation and cytoplasmic signals from cell surface receptors to the cell nucleus. The induction of MEK-ERK, which is associated with the proliferation of tumor cells, has been found in relevant head and neck squamous cell carcinoma cell lines and in vitro tissue culture models [21]. Within the MAPK pathway, MEK acts as a kinase that transfers signals from cell surface receptors and upstream kinases to downstream ERK [22]. Trametinib is an effective inhibitor of MEK1 and MEK2 and is capable of blocking RAF-dependent MEK phosphorylation, thus continuously inhibiting the activity of p-ERK1/2 [23]. Given these advancements in molecular biology and the rise of targeted therapy and precision medicine, there is an urgent need to develop new therapeutic approaches based on a deeper understanding of HNSC pathogenesis.

## Methods

### Experimental cell lines

This part of the experiment utilized four types of head and neck squamous cell carcinoma (HNSC) lines: the human tongue squamous carcinoma cell line Cal27, the human pharyngeal squamous carcinoma cell line Fadu, the human tongue squamous carcinoma cell line HN6, and the human laryngeal carcinoma cell line Tu686. Cal27 and Fadu were obtained from the National Biomedical Cell Bank (China), whereas HN6 and Tu686 were sourced from the Cell Bank of the Chinese Academy of Sciences Committee on Type Culture Collection. For *CHRNA5*, the primer sequences were as follows: Forward Primer: CCCAGGTTCTTGATCGGATG; Reverse Primer: GAACTGGTATTAATATATTTGCCCA. For *CES1*, the primer sequences were as follows: Forward Primer: ATCCACTCTCCGAAGGGCAACT; Reverse Primer: GACAGTGTCGTCTGTTCCTCCT.

Stable transfection strains of Cal27 and Tu686 cells were constructed using lentiviruses to knockdown *CHRNA5*. The target lentiviral vector was GV493, component sequence: hU6-MCS-CBh-gcGFP-IRES-puromycin; control number: CON313; Target Seq GGACTTGCAATATCTCAATTG; control insert sequence: TTCTCCGAACGTGTCACGT. This study was approved by the Ethics Committee of the Shanghai General Hospital of Shanghai Jiao Tong University School of Medicine.

### Establishment of cell model for evaluating cell migration-related functions

A head and neck squamous carcinoma cell model was established to expose cells to nicotine using the CCK-8 assay to evaluate the proliferation capabilities of Cal27, Fadu, HN6, and Tu686 cells after nicotine exposure. Cell scratch assays and Transwell experiments were conducted to quantitatively assess the migration and invasion capabilities of cells following nicotine exposure and *CHRNA5* knockdown. Western blot was used to quantify the expression levels of epithelial-mesenchymal transition (EMT)-related markers in cells exposed to nicotine following *CHRNA5* knockdown. The expression of nAChR mRNA and protein was detected using qRT-PCR and western blot. Immunofluorescence staining was used to examine the expression levels of CHRNA5 and Ki-67 post-*CHRNA5* knockdown, as well as the subcellular localization of CHRNA5 in tumor cells. Clonogenic assays were used to evaluate the clonogenic dependency and proliferation capabilities of cells post-*CHRNA5* knockdown. Using sh-*CHRNA5* Cal27 and Tu686 cell lines exposed to 10 μmol/L nicotine, samples were collected for RNA sequencing. qRT-PCR and western blot validated the transcription of *CES1* mRNA and its protein translation post-*CHRNA5* knockdown. The interaction between CHRNA5 and CES1 was investigated using molecular docking and molecular dynamics simulations, along with co-immunoprecipitation experiments and immunofluorescence colocalization. Differentially expressed genes were subjected to pathway enrichment analysis, and western blot experiments validated the expression of MEK/ERK proteins and their phosphorylation levels. The antibodies used were MEK/p-MEK (CST, 8727T/9154T) and ERK/p-ERK (CST, 4695T/4370T) for MEK and ERK proteins, respectively. For CES1, antibodies from Abcam (ab53008), Proteintech (67079-1-Ig), and ABclonal (A11478) were used. CHRNA5 antibodies were obtained from Abcam (ab259859) and Proteintech (66363-1-Ig).

### Molecular docking and molecular dynamics simulation

Protein Docking: The amino acid sequences of CHRNA5 and CES1 were retrieved from the UniProt protein database (https://www.uniprot.org/). Protein structure files for CHRNA5 (P30532) and CES1 (P23141) were obtained from the Alphafold database (https://alphafold.ebi.ac.uk/). Homology modeling based on amino acid sequences was performed using the I-TASSER database (https://seq2fun.dcmb.med.umich.edu//I-TASSER/). The GRAMM-X online tool (https://gramm.x3dna.org/) was used to dock CHRNA5 (3742 atoms) as the receptor and CES1 (4408 atoms) as the ligand. Protein-protein interactions were explored and visually analyzed using Pymol (version 2.4) and PDBePISA (https://www.ebi.ac.uk/pdbe/pisa/). Small-molecule docking: The SDF file of the MEK/ERK pathway inhibitor PD98059 was downloaded from the PubChem database (https://pubchem.ncbi.nlm.nih.gov/). AutoDock software was used for the dehydration and hydrogenation of small molecules as well as for identifying rotatable bonds. Similarly, it processed the CHRNA5 protein structure to prepare it for docking with the small molecules, PD98059 and CHRNA5.

Molecular dynamics simulations were performed using Gromacs version 2022.3. Preprocessing of the small molecule included the addition of the GAFF force field using AmberTools22 and the hydrogenation and RESP potential calculation of the small molecule using Gaussian 16 W. After the simulation, the trajectory data were thoroughly analyzed using the built-in analysis tools of the simulation software. This analysis included the calculation of root mean square deviation (RMSD), root mean square fluctuation (RMSF), protein gyration radius (Rg), and solvent-accessible surface area (SASA). These calculated results provide important foundational data for subsequent detailed analysis.

### Nude mouse tumorigenesis experiment

Initially, 5–6-week-old nude mice were selected for this study. The experiment proceeded with the preparation of Cal27 cells, which were stably transfected with sh-*CHRNA5* lentivirus and a control group; these cells were revived, cultured, and resuspended at a density of 5 × 10^7^ cells/mL. Subsequently, the mice were randomly divided into two experiments: Experiment 1 comprising the NC (negative control) and SH (sh-*CHRNA5*) groups, and Experiment 2, which included the SH group and the SH + NIC (SH + nicotine) group, with eight mice in each experiment, distributed into subgroups of 4. For the injection phase, a 100 μL cell suspension was subcutaneously injected into the right axilla of each mouse.

In the post-injection care phase, the SH + NIC group mice were exposed to smoke twice daily, with no less than 4 h between sessions, following a regimen mimicking the acute model of Chronic Obstructive Pulmonary Disease (COPD). This involved at least 5 days a week, with each session lasting 1 h under smoke conditions that adhered to ISO/CIR standards, employing no less than four cigarettes per animal per session, ensuring a smoke concentration greater than 350 mg/m^3^. To potentially shorten the smoking modeling period, lipopolysaccharide (LPS) was injected on days 15th and 45th days. The total smoking duration was extended to 35 days.

Tumor development was closely monitored throughout the experiment. Starting on day 7 post-injection, the length and width of each tumor were measured using a caliper, and measurements were recorded every subsequent 7 days. On the 35th day, the mice were euthanized by cervical dislocation, after which the tumor length and width were measured to calculate the volume (mm^3^ = 1/2*LW*^2^). Finally, the tumors were excised, weighed, and photographed for further analysis.

### Paraffin embedding and immunohistochemistry

Paraffin blocks, including those from nude mice tumorigenesis experiments (NC, SH, and SH + NIC groups) and HNSC tissues (laryngocarcinoma and hypopharyngeal carcinoma and their controls), were sectioned into 4–5 μm thick slices and mounted on slides. This setup also encompasses laryngocarcinoma and normal tissue combination microarray chips, featuring 29 squamous carcinoma cases and 10 normal tissues, with two-point sampling for one case. In the following section, the process includes drying, deparaffinization, rehydration, and then the immunohistochemistry phase. This phase starts with heat-induced antigen retrieval using sodium citrate buffer, followed by inactivation of endogenous enzymes, serum blocking to prevent non-specific binding, primary and secondary antibody incubations, and DAB staining for color development. After staining, sections were dehydrated, cleared, and sealed with neutral gum for preservation. Slide mounting, photography, and data quantification: The slides were sealed with neutral gum, observed, and photographed under a microscope. Staining quantification was performed using ImageJ software with the help of the IHC Profiler plugin to implement the Histochemistry Score (H-score). The H-score was calculated as (percentage of weak staining × 1) + (percentage of moderate staining × 2) + (percentage of strong staining × 3), with higher scores indicating stronger overall positive intensity.

### Statistical analysis

GraphPad Prism 10 software was used for statistical analysis and graphing. Student’s t-test was employed to analyze differences between groups, with a *p*-value of less than 0.05 considered statistically significant. Additionally, R software (version 3.6.1), available on the R Project website(https:/www.r-Project.org/), was utilized for statistical analyses. For data visualization, the “ggstatsplot” package in R was used, which can be found at CRAN R-project (https://CRAN.R-project.org/package=ggstatsplot). All the groups in this study were open experiments.

## Results

### Nicotine exposure promotes the metastasis of head and neck squamous carcinoma cells

**Fig. 1:**
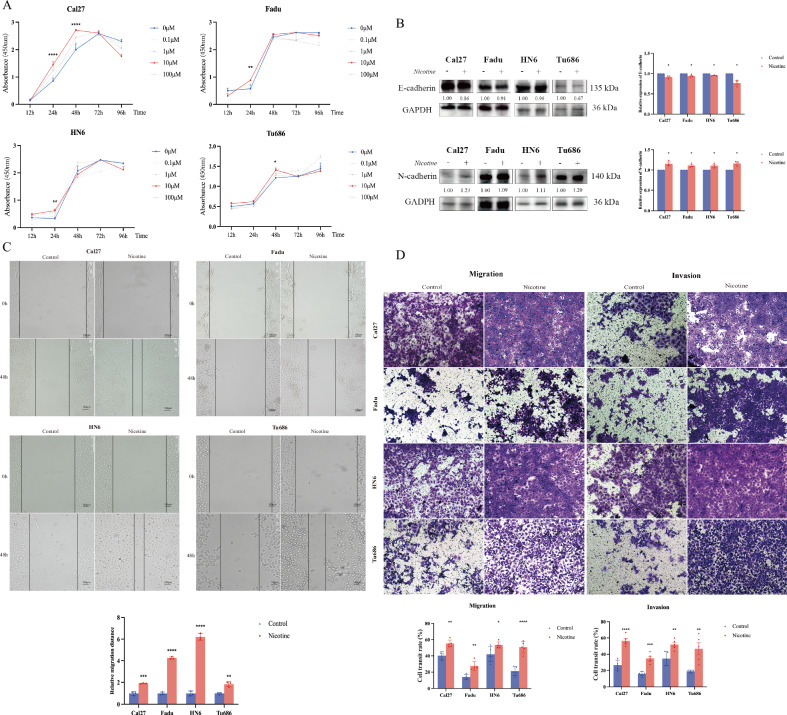
Nicotine exposure promotes the metastasis of HNSC cells. **A** Impact of different concentrations of nicotine on the proliferation capacity of Cal27, Fadu, HN6, and Tu686 cells; **B** Exposure to 10 μmol/L nicotine promotes EMT ability in the cells. Nicotine exposure significantly decreases the expression of E-cadherin and N-cadherin; **C** Exposure to 10 μmol/L nicotine promotes scratch migration in the cells. Nicotine exposure enhances migration in the cells; **D** Exposure to 10 μmol/L nicotine enhances migration and invasion capabilities of cells. Nicotine exposure promotes the migration and invasion ability of cells. (* *p* < 0.05, ** *p* < 0.01, *** *p* < 0.001, **** *p* < 0.0001).

To investigate the effect of nicotine on the proliferative capacities of the four cell lines, we cultured these cell lines at different final concentrations (100 μmol/L, 10 μmol/L, 1 μmol/L, 0.1 μmol/L, and 0 μmol/L) and observed them at 12 h, 24 h, 48 h, 72 h, and 96 h, respectively. The results revealed that a nicotine concentration of 10 μmol/L significantly promoted cell proliferation, showing a significant difference compared with the control group (*p* < 0.05). Specifically, between 24 h to 48 h after drug treatment, cells exhibited significant proliferative differences, subsequently entering a plateau phase. Cal27 cells demonstrated obvious proliferative differences at both 24 h and 48 h (*p* < 0.01), while Fadu cells showed significant differences at 24 h (*p* < 0.05). HN6 cells also exhibited noticeable proliferative differences at 24 h (*p* < 0.01), while Tu686 cells showed significant proliferative differences at 48 h (*p* < 0.01) (Fig. 1A).

To investigate the effects of nicotine exposure at a concentration of 10 μmol/L on the migratory capabilities of head and neck squamous carcinoma cells, we conducted in vitro wound healing assays and analyzed the relative migration distances of cells under serum-free conditions for 48 h. The results indicated that, compared to the control group, nicotine treatment significantly enhanced the migration of Cal27, FaDu, HN6, and Tu686 cells towards the wound area, improving their “healing” capacity, with this difference being statistically significant (*p* < 0.01) (Fig. 1C). Through Transwell migration and invasion assays, we explored the impact of nicotine on the migratory and invasive abilities of head and neck squamous carcinoma cells. In Transwell assays, exposure to 10 μmol/L nicotine significantly promoted the migration and invasion of Cal27, Fadu, HN6, and Tu686 cells compared to the control group (*p* < 0.05, Fig. 1D). The increased number of cells on the bottom filters of the chambers, with or without a matrix gel, indicated that nicotine exposure significantly enhanced the cell migration and invasion capabilities. Using western blot analysis, we detected changes in the expression of the epithelial-mesenchymal transition (EMT)-related proteins E-cadherin and N-cadherin after exposure to 10 μmol/L nicotine. The results showed that compared to the control group, the expression of E-cadherin was downregulated and the expression of N-cadherin was upregulated after nicotine exposure, with these differences being statistically significant (*p* < 0.05, Fig. 1B).

### Nicotine exposure promotes the transcription and translation of the nicotinic acetylcholine receptor (nAChR) gene CHRNA5

**Fig. 2:**
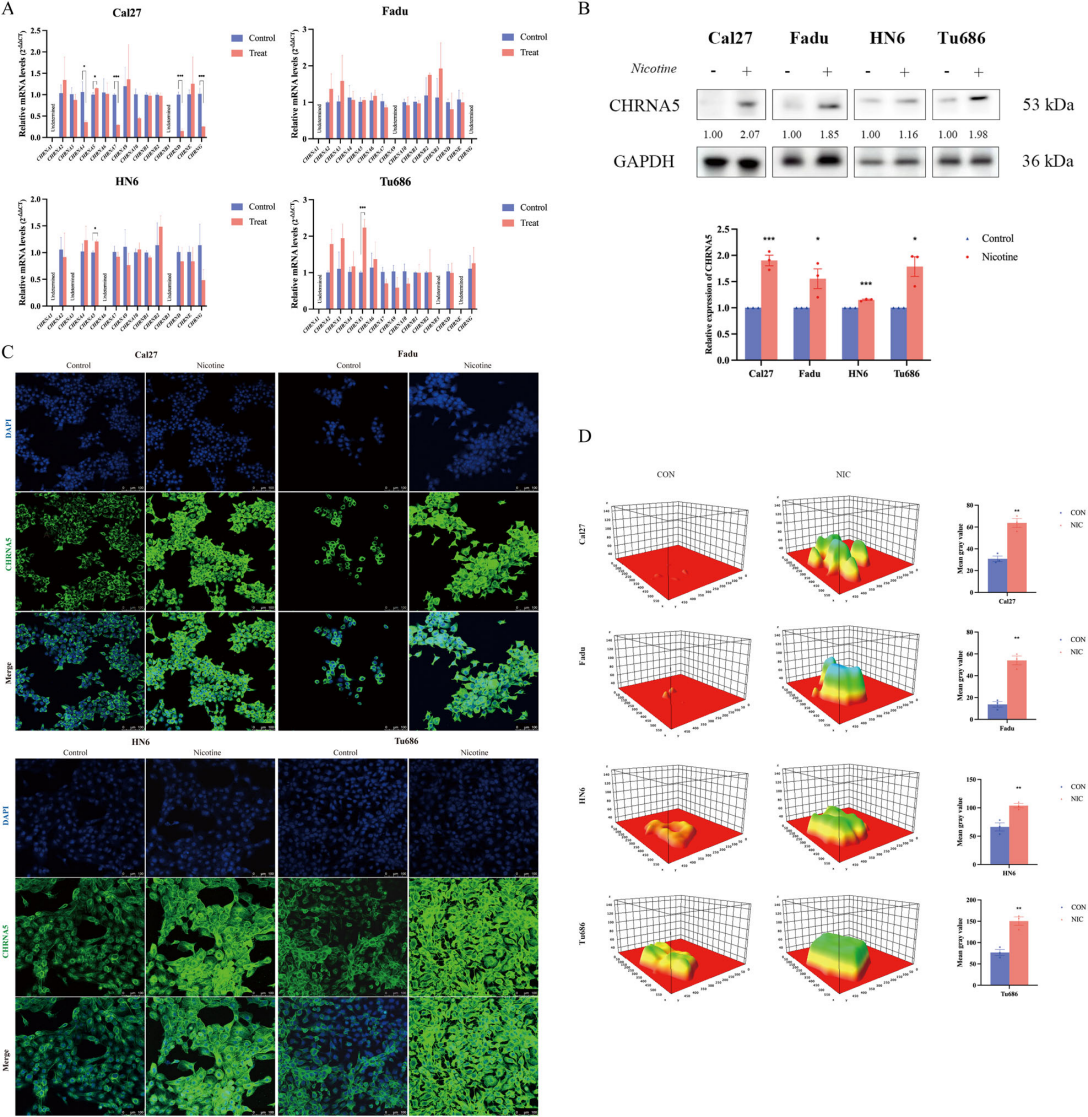
Nicotine exposure promotes the transcription and translation of *CHRNA5.* **A** 10 μmol/L nicotine significantly increases the mRNA expression levels of *CHRNA5* in Cal27, Fadu, HN6, and Tu686 cells; **B** 10 μmol/L nicotine significantly increases the protein expression levels of CHRNA5 in the cells; **C** Expression of CHRNA5 protein in the cells exposed to 10 μmol/L nicotine observed under laser confocal microscopy. CHRNA5 protein is primarily expressed in the cytoplasm and cell membrane, with nicotine significantly increasing the protein expression levels of CHRNA5 in the cells; **D** Quantitative analysis of CHRNA5 protein expression in the cells exposed to 10 μmol/L nicotine under laser confocal microscopy. Nicotine significantly increases the protein expression levels of CHRNA5 in the cells. The quantitative representation of average fluorescence intensity in the 3D graph is depicted using a rainbow color spectrum, where red indicates the lowest expression and purple indicates the highest expression. (*p < 0.05, ** *p* < 0.01, *** *p* < 0.001).

Using qRT-PCR, we systematically investigated the specific impact of nicotine exposure on the transcription levels of nicotinic acetylcholine receptor (nAChR) family genes. We examined the mRNA expression of 15 nAChR genes (including *CHRNA1, CHRNA2, CHRNA3, CHRNA4, CHRNA5, CHRNA6, CHRNA7, CHRNA9, CHRNA10, CHRNB1, CHRNB2, CHRNB3, CHRND, CHRNE, CHRNG*) in Cal27, Fadu, HN6, and Tu686 cells after exposure to 10 μmol/L nicotine. We found that in Cal27, HN6, and Tu686 cells, the expression of the *CHRNA5* gene was significantly upregulated (*p* < 0.05), whereas in FaDu cells, although there was an increase in expression, the difference was not statistically significant (Fig. 2A).

Additionally, through western blot experiments, we investigated the translational level of nAChR genes. The results indicated a significant increase in CHRNA5 protein expression in Cal27, Fadu, HN6, and Tu686 cells after nicotine exposure (Fig. 2B). Moreover, using an inverted fluorescence microscope and a laser scanning confocal microscope to observe immunofluorescence staining, we found that nicotine exposure significantly elevated the expression of CHRNA5 in head and neck squamous carcinoma cells. We also noted that CHRNA5 was primarily distributed in the cell membrane and cytoplasm (*p* < 0.05; Fig. 2C/D).

### Nicotine exposure reversal through knockdown of *CHRNA5* reduces migration and invasion of head and neck squamous carcinoma cells, EMT, and single-cell proliferative capacity

**Fig. 3:**
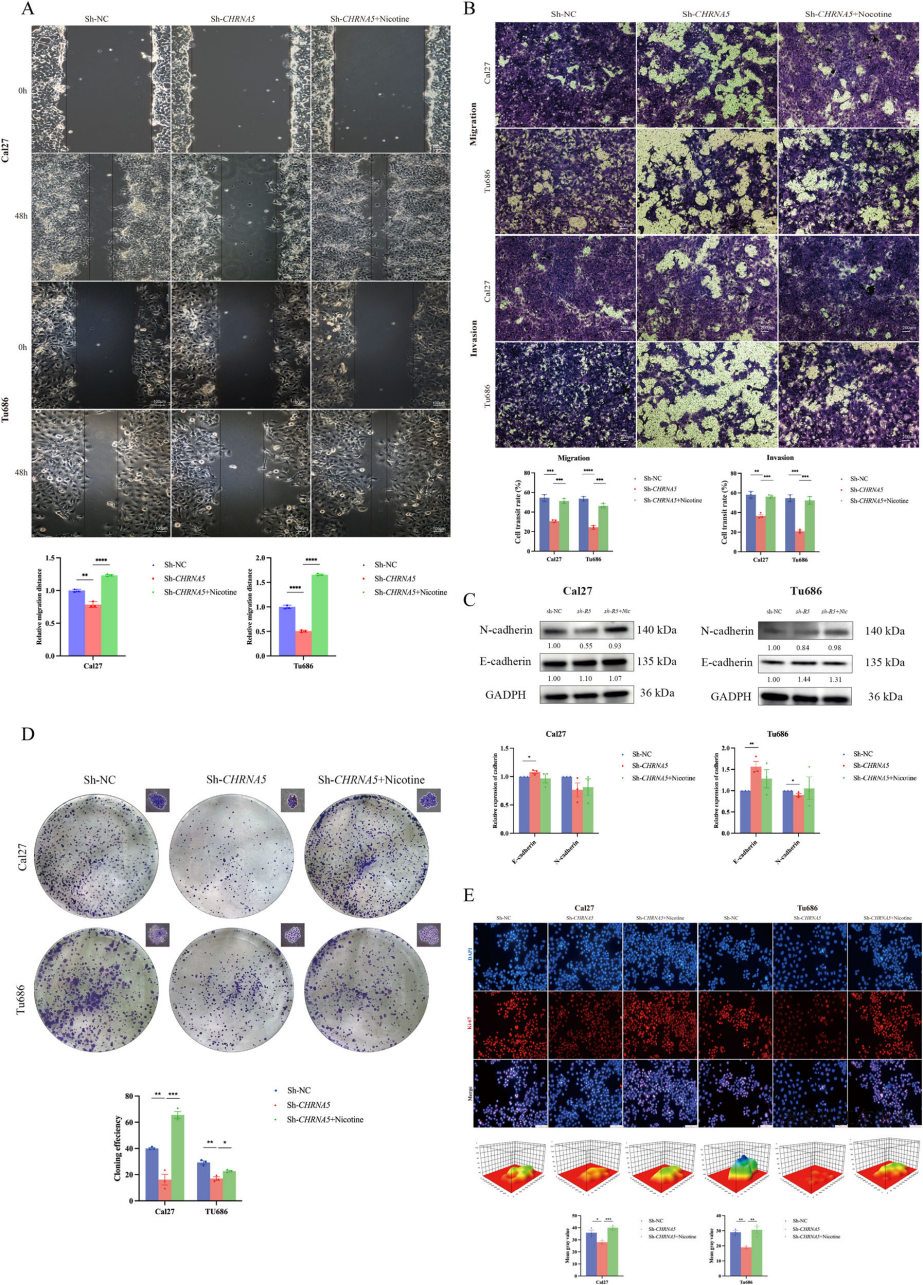
Nicotine exposure reversal through knockdown of *CHRNA5* reduces migration and invasion of HNSC cells, EMT, and single-cell proliferative capacity. **A** Knockdown of *CHRNA5* reduces scratch migration in head and neck squamous cell carcinoma cells, while nicotine exposure re-promotes migration in Cal27 and Tu686 cells; **B** Knockdown of *CHRNA5* reduces migration and invasion capabilities in Cal27 and Tu686 cells, while nicotine exposure re-promotes migration and invasion; **C** Knockdown of *CHRNA5* reduces EMT ability in Cal27 and Tu686 cells, while nicotine exposure re-promotes EMT ability; **D** Knockdown of *CHRNA5* reduces single-cell proliferation in Cal27 and Tu686 cells, while nicotine exposure re-promotes clonogenicity; **E** Knockdown of *CHRNA5* reduces Ki-67 protein expression in Cal27 and Tu686 cells, while nicotine exposure re-promotes Ki-67 expression. The quantitative representation of average fluorescence intensity in the 3D graph is depicted using a rainbow color spectrum, where red indicates the lowest expression and purple indicates the highest expression. (* *p* < 0.05, ** *p* < 0.01, *** *p* < 0.001, **** *p* < 0.0001).

To investigate the role of *CHRNA5* in the migration and invasion of head and neck squamous carcinoma cells, we successfully constructed sh-*CHRNA5* stable transfectants in Cal27 and Tu686 cells. Initially, we performed in vitro wound-healing assays to analyze the relative migration distance of the cells under serum-free conditions for 48 h. The results indicated that in both Cal27 and Tu686 cells, the migration capability towards the scratched area was reduced in the sh-*CHRNA5* group compared to that in the sh-NC group (*p* < 0.01). However, after nicotine exposure, the migration capability was restored and slightly enhanced, significantly improving cell “healing” ability (*p* < 0.0001) (Fig. 3A). Subsequently, through Transwell migration and invasion assays, we systematically explored the impact of *CHRNA5* on the migration and invasion abilities of head and neck squamous carcinoma cells. In the Transwell assays, compared to the sh-NC group, the sh-*CHRNA5* group showed significantly reduced migration and invasion abilities of Cal27 and Tu686 cells (*p* < 0.01). Notably, after nicotine exposure, the cell migration and invasion abilities were significantly restored (*p* < 0.001) (Fig. 3B). Western blot analyses were conducted to examine the changes in the expression of the EMT-related proteins E-cadherin and N-cadherin after *CHRNA5* knockdown. The results showed that, compared to the sh-NC group, the expression of E-cadherin was upregulated in the sh-*CHRNA5* group (*p* < 0.05); after nicotine exposure in the sh-*CHRNA5* group, the expression of E-cadherin showed a downward trend, although it was not statistically significant. In Tu686 cells, the expression of N-cadherin was significantly decreased in the sh-*CHRNA5* group, while in Cal27 cells, it showed a downward trend without statistical significance. In both cell types, after nicotine exposure in the sh-*CHRNA5* group, the expression of N-cadherin showed an upward trend, but it was not statistically significant (Fig. 3C).

To investigate the impact of *CHRNA5* on the clonogenic dependency and proliferative capability of individual head and neck squamous carcinoma cell populations (cell clusters formed through continuous in vitro proliferation over six generations or more), clonogenic assays were conducted. The results demonstrated a significant reduction in the clonogenic dependency and proliferative ability of individual cell populations in the sh-*CHRNA5* group compared with the sh-NC group in both Cal27 and Tu686 cells, as evidenced by a decrease in the clone formation rate (*p* < 0.01). Notably, nicotine exposure restored the clonogenic dependency and proliferative capacity of these cells, as indicated by an increase in the clone formation rate (*p* < 0.05) (Fig. 3D). Ki67, a nuclear protein associated with cell proliferation, is primarily located in the nucleolar periphery during the interphase of cell division and recruited to condensed chromosomes during mitosis. Typically, Ki67 is only expressed in proliferating cells. The expression level of this protein effectively reflects the proliferative status of cells. Therefore, immunofluorescence assays were performed on Cal27 and Tu686 cells to assess the abundance of the Ki-67 protein. The results showed that the sh-*CHRNA5* group exhibited a significant reduction in Ki-67 protein expression in both Cal27 and Tu686 cells (*p* < 0.05). Following nicotine exposure, a significant increase in Ki-67 protein expression was observed (*p* < 0.01) (Fig. 3E).

**Fig. 4:**
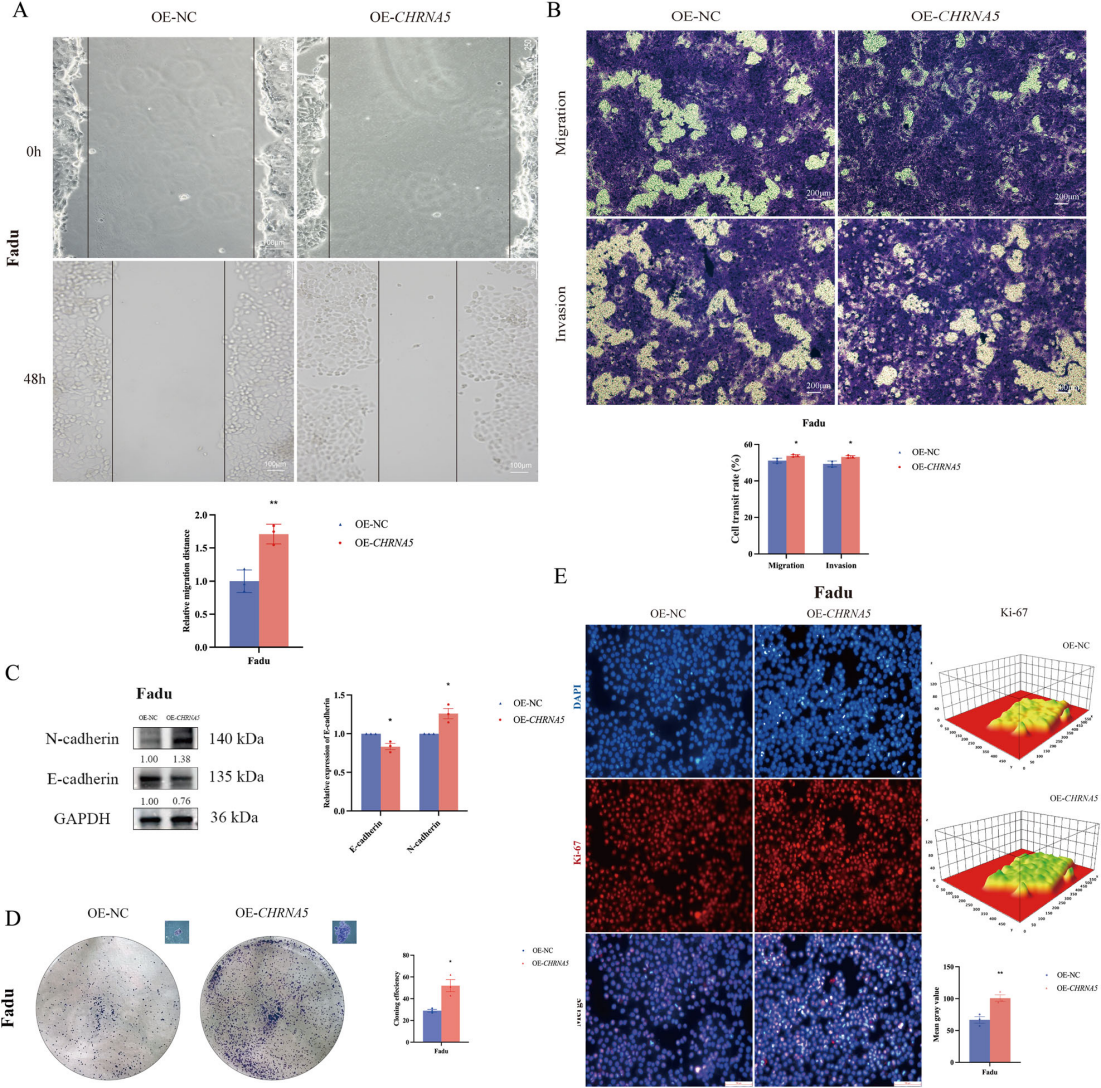
Overexpression of *CHRNA5* increases the migration and invasion, EMT and single-cell proliferative capacity of Fadu cells. **A** Overexpression of *CHRNA5* increases scratch migration of Fadu cells; **B** Overexpression of *CHRNA5* increases the migration and invasion of Fadu cells; **C** Overexpression of *CHRNA5* increases the EMT ability of Fadu cells; **D** Overexpression of *CHRNA5* increases the single-cell proliferative capacity of Fadu cells; **E** Overexpression of *CHRNA5* increases the Ki-67 protein expression of Fadu cells. The quantitative representation of average fluorescence intensity in the 3D graph is depicted using a rainbow color spectrum, where red indicates the lowest expression and purple indicates the highest expression. (* *p* < 0.05, ** *p* < 0.01, *** *p* < 0.001, **** *p* < 0.0001).

Correspondingly, we successfully established an OE-*CHRNA5* stable transfection cell line in Fadu cells. First, an in vitro wound healing assay was performed, and the results showed that, compared to the OE-NC group, the migration ability of Fadu cells toward the scratch area was significantly enhanced in the OE-*CHRNA5* group (*p* < 0.01) (Fig. 4A). Subsequently, the Transwell assay indicated that the migration and invasion abilities of Fadu cells were increased in the OE-*CHRNA5* group (*p* < 0.05) (Fig. 4B). We also analyzed the changes in the expression of EMT-related proteins E-cadherin and N-cadherin after *CHRNA5* overexpression by Western blot. The results showed that in the OE-*CHRNA5* group, E-cadherin expression was downregulated (*p* < 0.05), while N-cadherin expression was upregulated (*p* < 0.05) (Fig. 4C). Next, we conducted a colony formation assay, and the results showed that in the OE-*CHRNA5* group, the clonogenicity and proliferation ability of individual cell populations in Fadu cells increased, as evidenced by the elevated colony formation rate (*p* < 0.05) (Fig. 4D). Finally, an immunofluorescence assay was performed to assess the abundance of the Ki-67 protein. The results showed that Ki-67 protein expression was significantly elevated in the OE-*CHRNA5* group (*p* < 0.01) (Fig. 4E).

### Transcriptomics reveals differential gene expression after *CHRNA5* knockdown and Gene Ontology (GO) and Kyoto Encyclopedia of Genes and Genomes (KEGG) enrichment analysis of differential genes

**Fig. 5:**
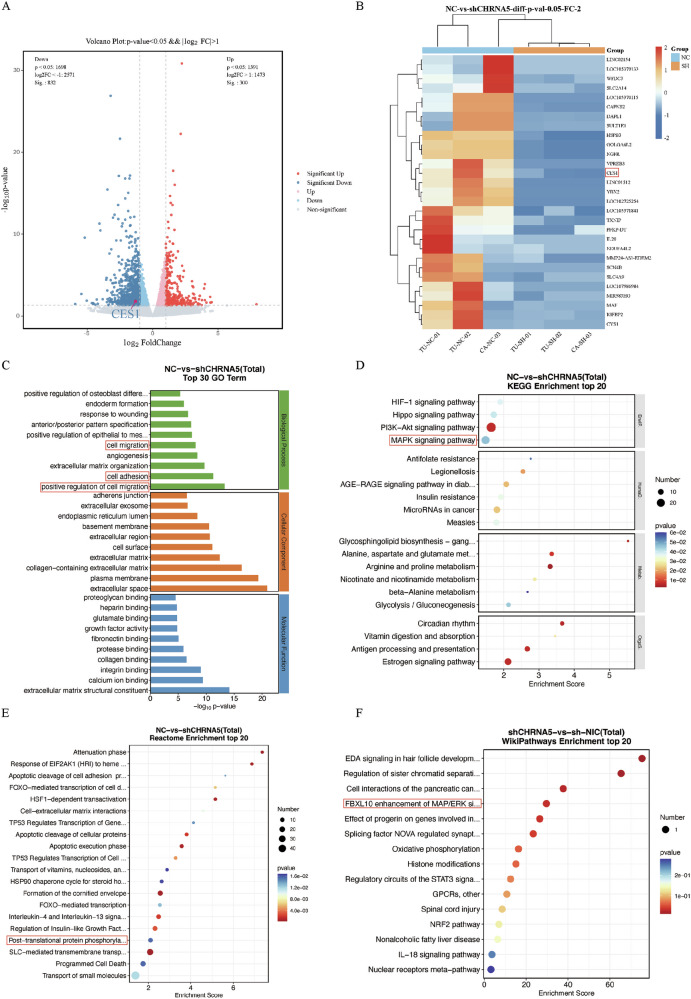
Transcriptomic sequencing with participant inclusion. **A** Volcano plot illustrating differential gene expression. Gray indicates genes with non-significant differential expression, while red and blue denote genes with significant differential expression. **B** Heatmap of differentially expressed genes. Red signifies relatively high expression of protein-coding genes, whereas blue represents relatively low expression of protein-coding genes. Enrichment analysis. **C** Differential gene enrichment analysis using Gene Ontology (GO) (NC-SH). **D** Enrichment analysis of differential genes using Kyoto Encyclopedia of Genes and Genomes (KEGG) pathways (NC-SH). The horizontal axis represents the Enrichment Score, while the vertical axis represents the categories. Bubble size reflects the number of differentially expressed protein-coding genes, and color changes from blue to red indicate the significance of enrichment *p*-values, with larger bubbles being more significant. **E** Enrichment analysis of differential genes using Reactome pathways (NC-SH). **F** Enrichment analysis of differential genes using WikiPathways (SH-NIC).

RNA integrity number (RIN) represents a numerical assessment of RNA integrity, ranging from 1 to 10, as provided by the Agilent Bioanalyzer for total RNA. A lower value indicates more significant degradation, with 10 representing the highest integrity. In this study, all nine samples (TU-NC-01, TU-SH-01, TU-NIC-01, TU-NC-02, TU-SH-02, TU-NIC-02, CA-NC-03, CA-SH-03, CA-NIC-03) had RIN values of 10, indicating high integrity and classifying them as Category A samples suitable for subsequent experiments. Reference-based transcriptome sequencing was performed for all nine samples, with each sample revealing gene expression data for more than 17,806 genes. After analyzing the differential gene expression data between the NC (control) and SH (*sh-CHRNA5*) groups, a total of 300 upregulated genes and 832 downregulated genes were identified. Among these genes, the expression level of the CES1 gene was significantly lower in the SH group, with statistically significant differences (*p* < 0.01) (Fig. 5A). Further cluster analysis of differential gene expression levels between the NC and SH groups showed that the expression level of CES1 was lower in the SH group (Fig. 5B).

In transcriptomics, Gene Ontology (GO) enrichment analysis for the NC and SH groups revealed the regulation of Biological Processes such as cell migration, cell adhesion, and positive regulation of cell migration (Fig. 5C). Concurrently, Kyoto Encyclopedia of Genes and Genomes (KEGG) enrichment analysis indicated that these regulations are associated with pathways, such as the MAPK signaling pathway (Fig. 5D). Additionally, Reactome enrichment analysis for the NC and SH groups showed associations with post-translational protein phosphorylation (Fig. 5E), while differential gene analysis between the SH and NIC groups in WikiPathways enrichment revealed relevance to FBXL10 enhancement of MAP/ERK signaling in diffuse large B-cell lymphoma (Fig. 5F).

### Molecular docking and co-immunoprecipitation analysis between *CHRNA5* and *CES1*

**Fig. 6:**
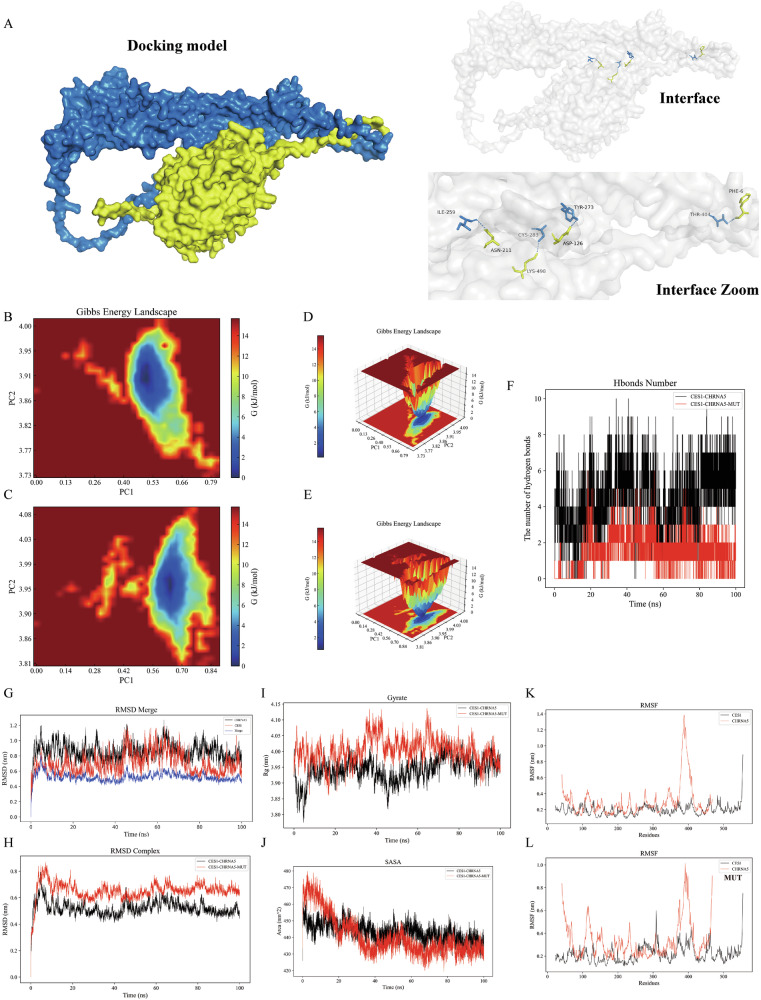
Gromacs molecular dynamics simulations of the CHRNA5-CES1 protein docking model and its mutant model. **A** Illustration of the original protein docking model, where CHRNA5 is shown in blue and CES1 in yellow, with hydrogen bond interactions represented by dashed lines. Free energy 2D and 3D landscapes of the original (**B**, **D**) and mutant (**C**, **E**) models, where the x-axis represents root-mean-square deviation (RMSD) and the y-axis represents the protein radius of gyration (Gyrate), both reflecting the protein energy states. The color spectrum indicates energy levels, with blue representing lower energy and red representing higher energy. A lower energy state indicates more stable protein binding, while the increased red energy area in the mutant model signifies higher instability. **F** Changes in hydrogen bonds between the original docking model and the mutant model. It demonstrates that the number of hydrogen bonds between proteins decreases in the mutant model (red), confirming the successful construction of the mutant model. **G** RMSD of the original docking model, with CHRNA5 in black, CES1 in red, and the overall docking model in blue, indicating that post-docking, the energy fluctuation range is small and remains in a low-energy state, suggesting stable binding. **H** RMSD changes of the original (black) and mutant (red) models, showing that the mutant model has greater energy fluctuation and remains in a high-energy state, indicating increased instability. **I** Gyrate changes in the original (black) and mutant (red) models, demonstrating that the mutant model exhibits greater energy fluctuation and remains in a high-energy state, signifying increased instability. **J** Solvent Accessible Surface Area (SASA) of the mutant model (red) fluctuates more widely, indicating increased instability. **K** Compared to the root mean square fluctuation (RMSF) of CHRNA5 and CES1 in the original docking model, the RMSF fluctuation range of both proteins increases in the mutant model, further indicating increased instability.

A rigid protein-protein docking study was conducted between CHRNA5 and CES1 using the GRAMM-X web server to perform free molecular docking with the receptor CHRNA5 (P30532) and ligand CES1 (P23141) data, which were homology-modeled using the I-TASSER database. After 3000 rounds of free matching, the best structure was obtained with an energy of −586 units. Pymol software was utilized to assemble the docking structure into a visualized complex, and specific hydrogen bonding sites compatible with docking in the PDBePISA database are shown in Table 1. The docking surface area between the two structures is 2049.3 Å^2^, with a binding energy of −26.3 kcal/mol. As shown in Fig. 6A, CHRNA5 and CES1 formed hydrogen bonds through amino acid residue sites (ILE 259-ASN 211, CYS 283-LYS 498, TYR 273-ASP 126, and THR 404-PHE 6).

**Table 1:** Protein-protein docking of the receptor CHRNA5 and ligand CES1 reveals hydrogen bonding positions.

NO.	CHRNA5	Dist.(Å)	CES1
1	ILE 259 [O]	3.38	ASN 211 [ND2]
2	CYS 283 [SG]	3.06	LYS 498 [NZ]
3	TYR 273 [OH]	3.44	ASP 126 [OD2]
4	THR 404 [OG1]	3.33	PHE 6 [O]

To further validate the original protein docking model, we mutated the predicted four amino acid sites to Alanine, which lacks a side chain, to form a mutant docking model [24, 25]. As shown in Fig. 6B/C/D/E, the 2D and 3D free energy landscape plots display the root-mean-square deviation (RMSD) on the x-axis and the radius of gyration (Gyrate) on the y-axis, both reflecting the protein energy states. The rainbow color scale represents energy levels, with blue indicating lower energy and red indicating higher energy. A lower energy state reflects more stable protein binding, while an increase in red areas in the mutant model indicates higher instability. As illustrated in Fig. 6F, compared to the original docking model, the number of hydrogen bonds between proteins in the mutant model is significantly reduced, confirming the successful construction of the mutant model. During the docking process between CHRNA5 and CES1, the RMSD values remained stable, and the combined RMSD was lower, indicating a smaller energy fluctuation range and a more stable binding in the original docking model (Fig. 6G). However, in the mutant model, the RMSD fluctuation range was larger and remained in a high-energy state, indicating increased instability (Fig. 6H). Similarly, the Gyrate fluctuation range in the mutant model was larger and remained in a high-energy state, indicating greater instability (Fig. 6I). The solvent-accessible surface area (SASA) also exhibited greater fluctuation in the mutant model, further indicating increased instability (Fig. 6J). Compared to the root-mean-square fluctuation (RMSF) of CHRNA5 and CES1 in the original docking model, the RMSF fluctuation range of both proteins was larger in the mutant model, suggesting increased instability (Fig. 6K, L). In conclusion, CHRNA5 and CES1 form a stable protein-protein docking model, but mutating their binding sites significantly reduces stability.

**Fig. 7:**
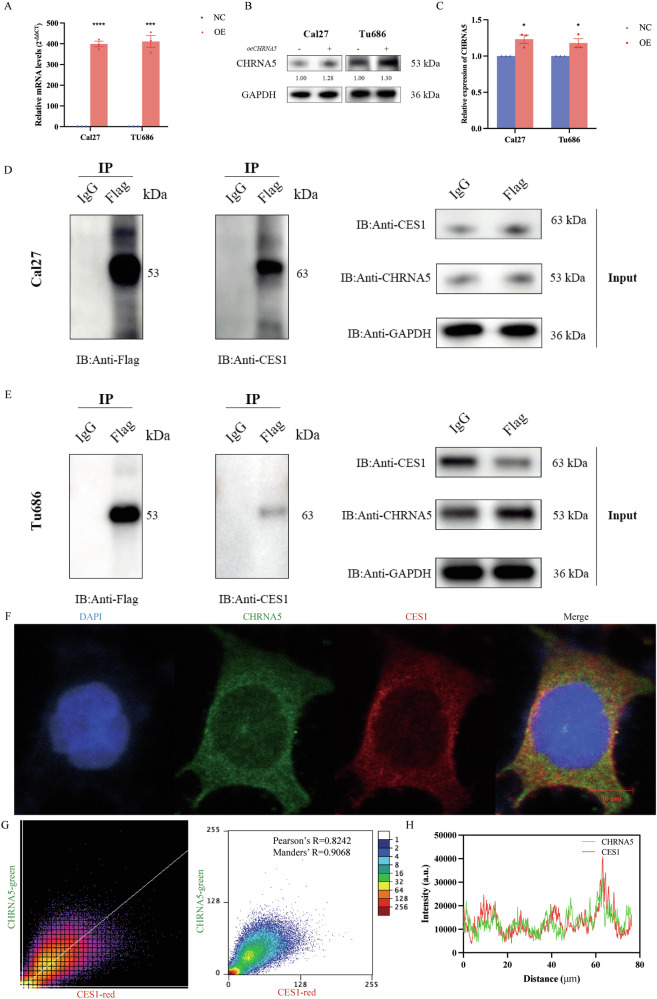
Immunoprecipitation of CHRNA5-CES1. **A** mRNA level validation after overexpression of CHRNA5. **B** Protein level validation after overexpression of CHRNA5. **C** Quantitative analysis of protein levels after overexpression of CHRNA5. **D** Immunoprecipitation validation of CHRNA5 and CES1 in Cal27 cells. **E** Immunoprecipitation validation of CHRNA5 and CES1 in Tu686 cells. **F** Confocal microscopy of triple-stained CHRNA5 (green), CES1 (red), and DAPI (blue) in Cal27 cells; **G** Scatter plot depicting the colocalization of CHRNA5 and CES1; **H** Line graph illustrating the colocalization of CHRNA5 and CES1.

To further confirm the interaction between CHRNA5 and CES1 proteins, a cell model overexpressing Flag-CHRNA5 was first constructed in Cal27 and Tu686 cell lines, and the specific results are shown in Fig. 7A–C. Results from qPCR and western blot experiments indicated that, compared to the control group, both mRNA and protein expression levels of CHRNA5 were significantly increased in these two cell lines (*p* < 0.05), demonstrating the successful construction of the cell model overexpressing Flag-CHRNA5. Subsequently, co-immunoprecipitation experiments were conducted in which CHRNA5 was tagged with FLAG. This allowed for the detection of CHRNA5 by identifying the FLAG tag and directly detecting CHRNA5. From the bands in the input group, it is clear that protein samples from both Cal27 and Tu686 cells contained CHRNA5 and CES1. In the IP IgG group, the bands were essentially not displayed, indicating that the results of the negative control group were normal. After overexpressing Flag-CHRNA5 in Cal27 and Tu686 cells, IP was performed using magnetic beads conjugated with anti-Flag antibody. Western blot analysis using antibodies against FLAG and CES1 showed bands at the positions of CHRNA5 and CES1, explicitly indicating an interaction between CHRNA5 and CES1 (Fig. 7D, E).

Immunofluorescence colocalization analysis of CHRNA5 and CES1 was also conducted, as shown in Fig. 7F–H, with triple staining in Cal27 cells: CHRNA5 (green), CES1 (red), and DAPI (blue). Quantitative analysis of the data revealed a Pearson’s correlation coefficient of 0.8242 and a Manders’ overlap coefficient of 0.9068 for the co-precipitation of CHRNA5 and CES1, indicating a high degree of colocalization and a high level of authenticity in the relationship between the two channels. These analytical results support a deeper understanding of the multifaceted relationship between CHRNA5 and CES1, revealing their complex and diverse associations from multiple perspectives, including physical, co-expression, and genetic interactions.

### Verification at the cellular level of the mechanism by which CHRNA5 regulates CES1 expression through the MEK/ERK pathway

The enrichment analysis shown in Fig. 5 revealed that the knockdown of *CHRNA5* might be associated with the MEK/ERK pathway. To further verify this finding, western blot experiments were conducted in the Cal27 and Tu686 cell lines. The MEK/ERK pathway inhibitor PD98059 was chosen as a tool for the experiments. PD98059 is an effective and selective inhibitor of MEK with an IC50 of 5 μM. It functions by binding to the inactive form of MEK, preventing the activation of MEK1 and MEK2 by upstream kinases, and acts as an inhibitor of the ERK1/2 signaling pathway. As shown in Supplementary Fig. S1, the effect of the small molecule PD98059 on the protein structures of CHRNA5 and CES1 was analyzed. It was found that out of 100 docking attempts between CHRNA5 and CES1, optimal docking resulted in only one hydrogen bond formation (THR 130 and SER 305), indicating weak binding and minimal effect.

**Fig. 8:**
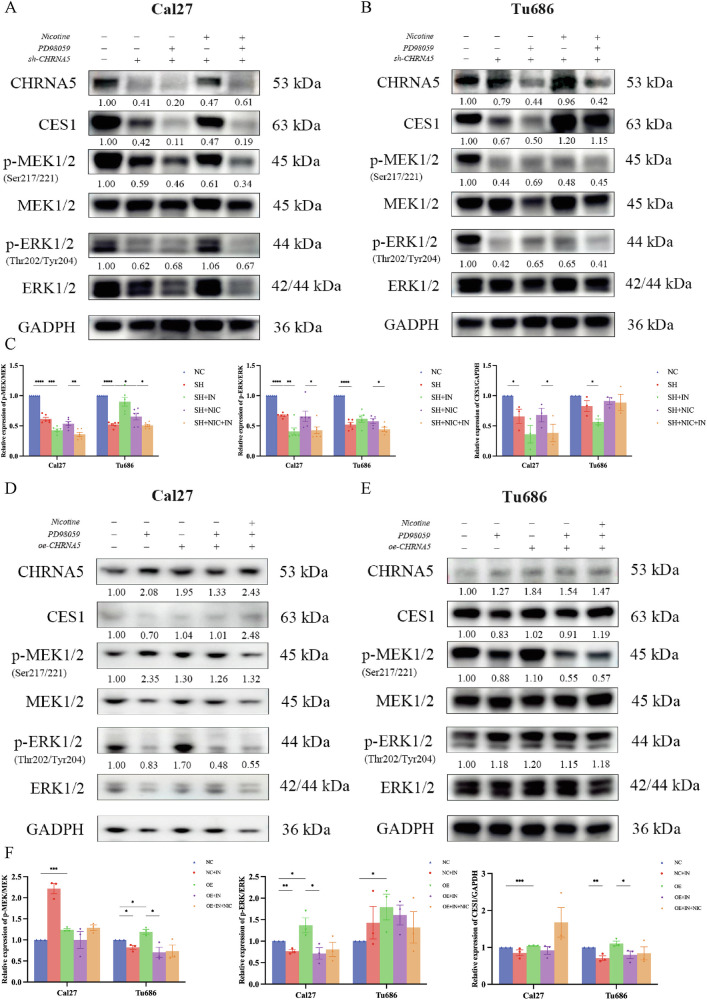
Cellular knockdown of *CHRNA5* influences CES1 expression through the MEK/ERK pathway. **A** Cal27 cells. **B** Tu686 cells. **C** Quantitative analysis of p-MEK/MEK, p-ERK/ERK, and CES1/GAPDH ratio. Cellular overexpression of *CHRNA5* affects CES1 expression through the MEK/MEK pathway. **D** Cal27 cells. **E** Tu686 cells. **F** Quantitative analysis of p-MEK/MEK, p-ERK/ERK, and CES1/GAPDH ratio.

The experimental design was divided into five groups: NC (control group), SH (knockdown group), SH + IN (knockdown + inhibitor group), SH + NIC (knockdown + nicotine group), and SH + NIC + IN (knockdown + nicotine + inhibitor group). The results showed that activated MEK (p-MEK/MEK) significantly decreased with the knockdown of *CHRNA5* (all *p* < 0.0001). PD98059 was able to further reduce p-MEK/MEK in the SH group (Cal27, *p* < 0.001), whereas nicotine was able to reverse the reduction of p-MEK/MEK in the SH group (Tu686, *p* < 0.05). PD98059 also inhibited the nicotine-induced increase in p-MEK/MEK (Cal27, *p* < 0.01; Tu686, *p* < 0.05). Similarly, activated ERK (p-ERK/ERK) significantly decreased with the knockdown of *CHRNA5* (all *p* < 0.0001). PD98059 was able to further reduce p-ERK/ERK compared to the SH group (Cal27, *p* < 0.01), but nicotine did not significantly reverse the reduction of p-ERK/ERK in the SH group. PD98059 inhibited nicotine-induced increase in p-ERK/ERK (all *p* < 0.05). Additionally, CES1 decreased with the knockdown of *CHRNA5* (Cal27, *p* < 0.05), and PD98059 was able to further reduce CES1 on the basis of the SH group (Tu686, *p* < 0.05), whereas nicotine did not significantly reverse the reduction of CES1 in the SH group. PD98059 inhibited nicotine-induced increase in CES1 (Cal27, *p* < 0.05) (Fig. 8A–C).

The following experiments utilized a cell model constructed to overexpress *CHRNA5* for further validation, which was divided into five groups: NC group (control group), NC + IN group (control + inhibitor group), OE group (overexpression group), OE + IN group (overexpression + inhibitor group), and OE + IN + NIC group (overexpression + inhibitor + nicotine group). The experiments demonstrated that the activation of MEK (p-MEK/MEK) increased with the overexpression of *CHRNA5* (Cal27, *p* < 0.001; Tu686, *p* < 0.05). PD98059 reduced p-MEK/MEK in the NC group (Tu686, *p* < 0.05) and reduced p-MEK/MEK in the OE group (Tu686, *p* < 0.05). Nicotine reversed the reduction in p-MEK/MEK levels in the OE + IN group, showing a trend but the difference was not statistically significant. Similarly, the activation of ERK (p-ERK/ERK) increased with the overexpression of *CHRNA5* (all *p* < 0.05). PD98059 reduced p-ERK/ERK levels in the NC group (Cal27, *p* < 0.01) and OE group (Cal27, *p* < 0.05). Nicotine reversed the reduction in p-ERK/ERK in the OE + IN group; however, the difference was not statistically significant. CES1 increased with the overexpression of *CHRNA5* (Cal27, *p* < 0.001), and PD98059 reduced CES1 in the NC group (Tu686, *p* < 0.01) and OE group (Tu686, *p* < 0.05). Nicotine reversed the reduction in CES1 in the OE + IN group, showing a trend but the difference was not statistically significant (Fig. 8D–F).

### Morphological comparison of tumor formation in nude mice and validation of CES1 regulation via the MEK/ERK pathway in tumorigenic tissues

**Fig. 9:**
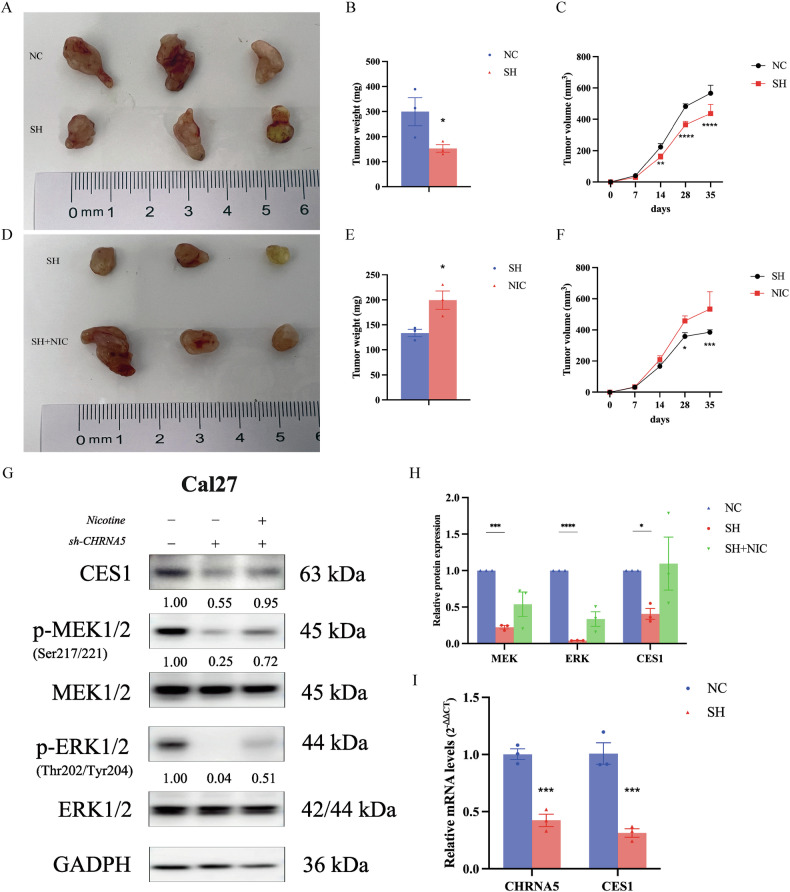
Nude mouse tumor morphology, PCR, and Western blot experiments. **A** Comparison of tumor tissues between the NC group and SH group. **B** Quantification of tumor weight in the NC group and SH group. **C** Changes in tumor volume in the NC group and SH group. **D** Comparison of tumor tissues between the SH group and SH + NIC group. **E** Quantification of tumor weight in the NC group and SH + NIC group. **F** Changes in tumor volume in the NC group and SH + NIC group. **G** Validation of MEK/ERK pathway and *CES1* protein expression changes in nude mouse tumors. **H** Quantitative analysis of MEK/ERK pathway and *CES1* protein expression in nude mouse tumors. **I** Comparison of *CHRNA5* and *CES1* transcription levels in tumor tissues between the NC group and SH group.

Tumorigenesis was induced in nude mice using sh-*CHRNA5* Cal27 stable transfectants, which were then divided into the NC group (control group), SH group (knockdown group), and SH + NIC group (knockdown + nicotine group). Tumor formation in each group of nude mice was measured every seven days. A comparison between the NC and SH groups revealed that from day 14 onwards, the tumor volume in the NC group was significantly larger than that in the SH group (*p* < 0.01). Comparing the SH group with the SH + NIC group, from day 28 onwards, the tumor volume in the SH + NIC group was significantly larger than that in the SH group (*p* < 0.05) (Fig. 9C, F). After tumor removal on day 35, both visual inspection and measurements showed that the tumor volume in the NC group was significantly larger than that in the SH group (weight, *p* < 0.05; volume, *p* < 0.0001), and the tumor volume in the SH + NIC group was significantly larger than that in the SH group (weight, *p* < 0.05; volume, *p* < 0.001) (Fig. 9A, B, D, E).

Subsequent western blot experiments on tumor tissue proteins revealed that activation of MEK (p-MEK/MEK) significantly decreased under *CHRNA5* knockdown (*p* < 0.001), whereas nicotine reversed the reduction of p-MEK/MEK in the SH group. Similarly, activated ERK (p-ERK/ERK) was also significantly decreased after *CHRNA5* knockdown (*p* < 0.0001); however, nicotine did not significantly reverse the trend of reduced p-ERK/ERK in the SH group. Additionally, the expression level of CES1 significantly decreased after *CHRNA5* knockdown (*p* < 0.05), and nicotine showed a trend towards reversing the reduction in CES1 in the SH group, although the effect was not significant (Fig. 9G, H). qRT-PCR validation of CES1 transcription levels in the NC and SH groups showed that CES1 mRNA expression levels significantly decreased with the knockdown of *CHRNA5* (*p* < 0.001), consistent with the protein expression trend (Fig. 9I).

### Pathological staining of nude mouse tumor tissue and human head and neck squamous carcinoma tissue

**Fig. 10:**
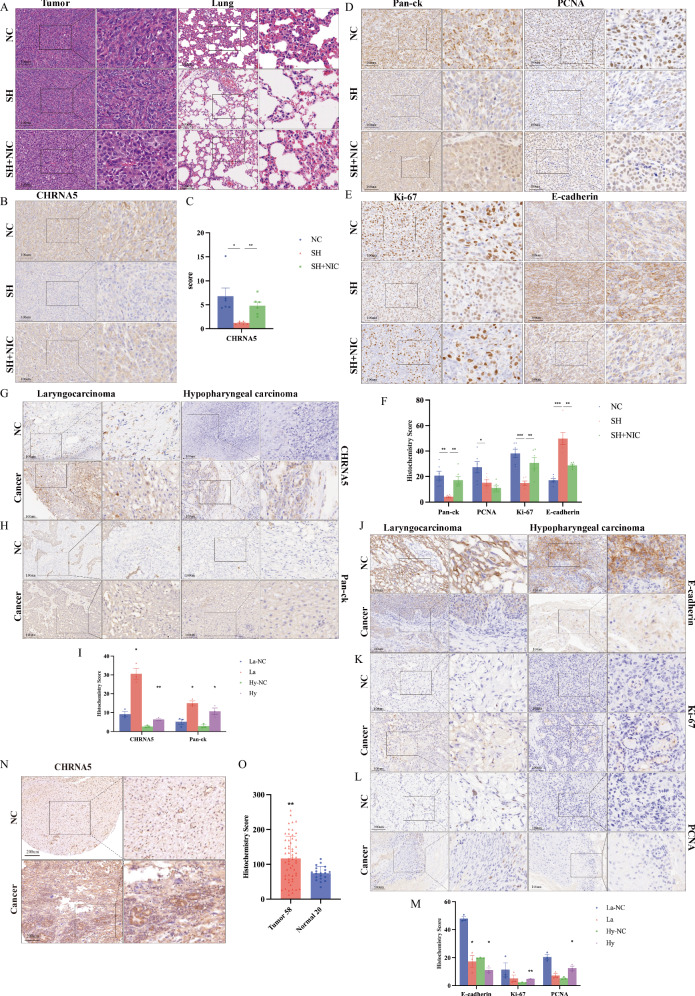
HE staining and immunohistochemistry of HNSC tissue. **A** Nude mouse tumor histopathology experiment (NC group, SH group, and SH + NIC group). HE staining of tumor tissues and lung tissues. **B** Immunohistochemical staining of CHRNA5. **C** Quantitative analysis of H-score. **D** Immunohistochemical staining of Pan-ck and PCNA. **E** Immunohistochemical staining of Ki-67 and E-cadherin. **F** Quantitative analysis of H-score. **G** Histopathology experiment on laryngocarcinoma and hypopharyngeal carcinoma tissues. Immunohistochemical staining of CHRNA5. **H** Immunohistochemical staining of Pan-ck. **I** Quantitative analysis of H-score. **J** Immunohistochemical staining of E-cadherin. **K** Immunohistochemical staining of Ki-67. **L** Immunohistochemical staining of PCNA. **M** Quantitative analysis of H-score. **N** Immunohistochemical experiments on laryngeal cancer tissue microarray. Immunohistochemical staining of CHRNA5. **O** Quantitative analysis of H-score.

Pathological section staining was performed on the tumor tissues of the three groups of tumor-bearing nude mice (NC, SH, and SH + NIC), as shown in Fig. 10A. HE staining of all three groups exhibited characteristics of undifferentiated carcinoma, with significant pleomorphism, densely arranged nucleoli observable within the nuclei, and multiple mitotic Fig. 10A. No obvious lung metastatic tissue was found in the HE staining of lung tissues.

As shown in Fig. 10B, C, the protein expression level of CHRNA5 in the SH group was lower than that in the NC group, with a significantly lower immunohistochemical score (H-score) than that in the NC group (*p* < 0.05), further confirming the successful construction of the Cal27 cell nude mouse tumor model. At the same time, the protein expression levels of Pan-ck (*p* < 0.01), PCNA (*p* < 0.05), and Ki-67 (*p* < 0.001) in the SH group were also lower than those in the NC group, with significantly lower H-score, while the protein expression level of E-cadherin in the SH group was higher than that in the NC group, with a significantly increased H-score (*p* < 0.001) (Fig. 10D–F). In the SH + NIC group, the protein expression level of CHRNA5 was higher than that in the SH group, with a significantly higher H-score than the SH group (*p* < 0.01). Nicotine reversed the expression level of CHRNA5, leading to increased protein expression levels of Pan-ck (*p* < 0.01), and Ki-67 (*p* < 0.01), with a significantly increased H-score. Simultaneously, the protein expression level of E-cadherin decreased, with a significantly reduced H-score (*p* < 0.01) (Fig. 10D–F).

Tissue samples were collected from patients with laryngocarcinoma treated surgically at our hospital and controls (3:3), as well as from patients with hypopharyngeal carcinoma and controls (3:3). Immunohistochemical staining revealed that the protein expression levels of CHRNA5 and Pan-ck in laryngocarcinoma and hypopharyngeal carcinoma were higher than those in the control group, with a significantly increased H-score (*p* < 0.05). Simultaneously, the protein expression levels of E-cadherin in laryngocarcinoma and hypopharyngeal carcinoma were lower than those in the control group, with a significantly reduced H-score (*p* < 0.05). In hypopharyngeal carcinoma, the protein expression levels of PCNA and Ki-67 were higher than those in the control group, with a significantly increased H-score (*p* < 0.05) (Fig. 10G–M). Subsequently, 29 patients with laryngocarcinoma and 10 normal control tissues were collected, with two tissue sites per case stained on an immunotissue microarray (58 cases of laryngeal cancer and 20 cases of normal control tissue). The results showed that CHRNA5 (*p* < 0.01) was highly expressed in patients with laryngocarcinoma, with a significantly increased H-score (Fig. 10N, O).

### The downstream gene CES1 expression in HNSC tissues, its association with the expression of proliferation-related proteins, prognosis, and single-cell sequencing expression localization

**Fig. 11:**
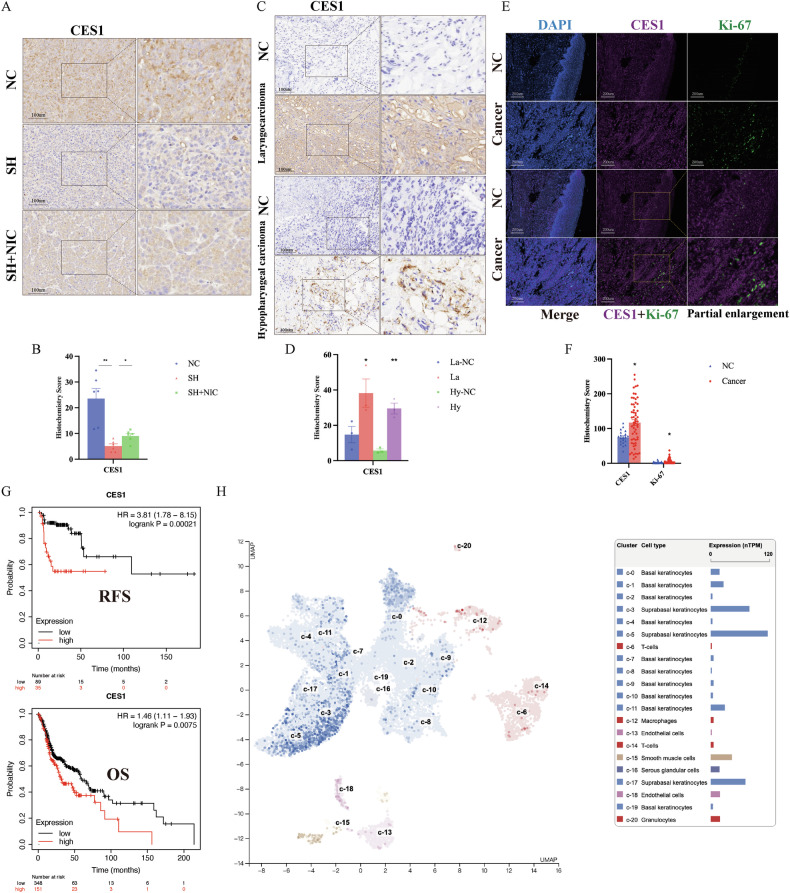
The downstream gene CES1 expression in HNSC tissues, its association with the expression of proliferation-related proteins, prognosis, and single-cell sequencing expression localization. **A**/**B** Immunohistochemical expression of CES1 in nude mouse tumor tissues and Quantitative analysis of CES1 H-score. **C**/**D** Immunohistochemical expression of CES1 in laryngocarcinoma and hypopharyngeal carcinoma tissues and Quantitative analysis of CES1 H-score. **E**/**F** Expression of CES1 and Ki-67 in laryngocarcinoma tissue microarray and Quantitative analysis of H-score. **G** Association between CES1 expression and prognosis of patients with HNSC. **H** Relationship between CES1 expression in tongue tissue and cluster cell types based on the Human Protein Atlas database.

As shown in Fig. 11A, B, CES1 protein decreases as the tumor shrinks following the intervention (*p* < 0.01) and increases with tumor growth after re-exposure to smoke intervention (*p* < 0.05). In laryngocarcinoma and hypopharyngeal carcinoma tissues, we found that CES1 is elevated in tumor tissues (*p* < 0.05, *p* < 0.01) (Fig. 11C, D). We further investigated the expression related to laryngocarcinoma tissue microarrays and found that as CES1 increases in laryngocarcinoma, the expression of the tumor proliferation and metastasis-related protein Ki-67 [26] also rises (*p* < 0.05) (Fig. 11E, F). We also collected data from the Kaplan-Meier Plotter database [27] on 499 patients with HNSC regarding CES1 expression and prognosis. It was found that high CES1 expression is closely associated with poorer overall survival (OS, *p* = 0.0075) and relapse-free survival (RFS, *p* = 0.00021) (Fig. 11G). We explored the cell type group-specific expression of CES1 in tongue tissue using single-cell sequencing data from The Human Protein Atlas database (https://www.proteinatlas.org/). CES1 was found to be primarily expressed in Suprabasal keratinocytes (C3, 5, 17), Basal keratinocytes (C0, 1, 11), and Endothelial cells (C18). Squamous cell carcinoma is often associated with keratinocytes and Endothelial cells [28–30]. Therefore, CES1 plays a significant role in tumor proliferation and metastasis (Fig. 11H).

## Discussion

Epidemiological investigations indicate that nicotine in cigarette smoke is significantly associated with the risk of various tumors [31, 32]. Many studies have shown that long-term exposure to cigarette smoke can affect immune function and health of smokers by affecting the innate and adaptive immune systems [33, 34]. Further research has found that cigarette smoke can induce EMT in lung cancer by activating signaling pathways such as STAT3, AKT, and NF-kB [35–37]. Nishioka [13] conducted a study on mouse lung epithelial cells (RLE) and mouse lung cancer cells (LKR) using a concentration of 0.5 μmol/L nicotine, exploring the effects of nicotine on cell growth and apoptosis. It was found that short-term nicotine exposure mildly activated mitotic signaling pathways (such as PKC, ERK, and Akt) and, to some extent, alleviated cisplatin-induced apoptosis. Shimizu’s research indicates [38]that nicotine at a concentration of 0.5 μmol/L can induce the phosphorylation of EGFR by activating the CHRNA7 pathway. Phosphorylated EGFR is transported from the cell membrane to the nucleus, activating the Akt and mTOR signaling pathways, thereby promoting the proliferation and migration of head and neck squamous carcinoma cells (HSC-2: derived from cervical lymph node metastasis, originating from squamous carcinoma of the oral floor; HSC-3, derived from cervical lymph node metastasis originating from tongue squamous carcinoma; OSC-19: derived from the primary site, originating from tongue squamous carcinoma; OSC-20, derived from cervical lymph node metastasis originating from tongue squamous carcinoma), ultimately leading to lymph node metastasis. Our study found that a nicotine concentration of 10 μmol/L significantly promoted the proliferation of head and neck cancer cells (Cal27, Fadu, HN6, and Tu686 cells) (*p* < 0.05). In vitro scratch assays showed that nicotine treatment significantly enhanced the migration of Cal27, Fadu, HN6, and Tu686 cells towards the scratched area, improving cell “healing” capability. In the Transwell migration and invasion assays, nicotine exposure significantly promoted the migration and invasion capabilities of Cal27, Fadu, HN6, and Tu686 cells (*p* < 0.05). Western blot results indicated that nicotine exposure led to upregulation of E-cadherin expression and downregulation of N-cadherin expression (*p* < 0.05).

The pathophysiological effects of nicotine and its derivatives are primarily mediated by nAChRs in target cells, especially in lung cancer, through sensitive subtypes of nAChRs, such as α7 (CHRNA7) [39, 40]. The interaction of nicotine with nAChR has been found to increase oxidative stress and activate multiple signaling pathways, such as NF-κB and MAPK, thereby regulating tumor progression, growth, and metastasis [41–43]. Through qRT-PCR experiments, we found that the mRNA expression of *CHRNA5* was significantly upregulated in Cal27, HN6, and Tu686 cells exposed to nicotine (*p* < 0.05). Western blot experiments showed that the expression of CHRNA5 protein was significantly increased in Cal27, FaDu, HN6, and Tu686 cells after exposure to nicotine (*p* < 0.05). Immunofluorescence staining revealed that nicotine exposure significantly increased the expression level of CHRNA5 in head and neck squamous carcinoma cells (*p* < 0.05), and CHRNA5 was mainly distributed in the cell membrane and cytoplasm. Knockdown of *CHRNA5* reduced migration, invasion, single-cell colony dependence, and proliferative abilities of Cal27 and Tu686 cells, and nicotine exposure reversed this trend.

Tumor cells must adapt to the nutrient-poor tumor microenvironment (TME) to survive, proliferate, and metastasize [44]. The high metabolic demands of rapidly growing tumor cells, combined with inefficient blood supply from abnormal vasculature, reduce nutrient availability, such as glucose, in solid tumors [45]. This metabolic stress is worsened by driver mutations that limit the cells’ response to stress while locking them into biosynthetic pathways [46]. Tumor clones that overcome this gain a growth advantage, promoting malignant progression, EMT, and metastasis [47]. NF-κB transcription factors, key regulators of immunity and inflammation, drive tumorigenesis, recurrence, and therapy resistance by upregulating genes that prevent apoptosis and coordinate inflammation in the TME [48]. Carboxylesterase 1 (*CES1*) is an NF-κB-regulated lipase that links inflammation and lipid metabolism, supporting the adaptation of progressive colorectal cancer (CRC) to energy stress. By enhancing fatty acid oxidation and preventing triglyceride toxicity, *CES1* can promote tumor initiation, progression, and metastasis, and elevated *CES1* expression is associated with poor prognosis. NF-κB-driven *CES1* expression has been observed to be related to tumor cachexia and inflammation [49]. Research [50] found that melatonin effectively inhibits lipid accumulation in prostate cancer cells by regulating the epigenetic aspects of *CES1*. This process enhances endoplasmic reticulum stress, promotes apoptosis, and reduces androgen synthesis, thereby preventing the progression and metastasis of prostate cancer. In hepatocellular carcinoma (HCC), suppression of *CES1* leads to a significant reduction in lipid signaling molecules of PPARα/γ, with a notable downregulation of the PPARα/γ target gene *SCD*, which is associated with tumor metastasis, recurrence, and chemoresistance. Disrupting lipid signaling by targeting the *CES1*-PPARα/γ-*SCD* axis enhances the sensitivity of HCC cells to cisplatin treatment. The use of cisplatin in combination with a *CES1* inhibitor effectively slows the growth of HCC xenograft tumors in NU/J mice [51]. In this study, sh-*CHRNA5* Cal27 and sh-*CHRNA5* Tu686 cell lines were treated with 10 μmol/L nicotine exposure followed by transcriptome sequencing. Differential gene analysis between the NC group and SH group revealed that the expression level of *CES1* was significantly lower in the SH group (*p* < 0.01), as shown by heatmap analysis, indicating reduced expression levels of *CES1* in the SH group. Our qRT-PCR results demonstrated that *CHRNA5* knockdown significantly reduced the expression of *CES1* in both cell lines (*p* < 0.05). Western blot analysis confirmed that CES1 protein expression was significantly decreased following *CHRNA5* knockdown (*p* < 0.05). These experimental outcomes further support the conclusions drawn from omics studies, indicating a significant regulatory effect of *CHRNA5* knockdown on the expression of *CES1*. Molecular docking and molecular dynamics simulations were conducted between CHRNA5 and CES1 to calculate RMSD, RMSF, Rg, and SASA, which indicated the formation of a stable protein docking model between CHRNA5 and CES1. Immunoprecipitation experiments demonstrated an interaction between CHRNA5 and CES1, and immunofluorescence colocalization analysis confirmed a strong colocalization distribution relationship between them.

**Fig. 12:**
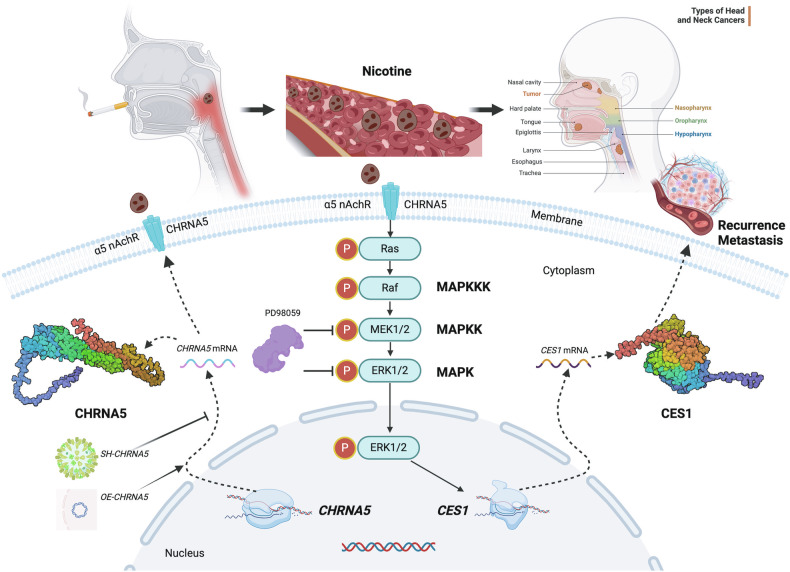
Graphical abstract. Mechanism diagram of nicotine exposure activating *CHRNA5* via MEK/ERK pathway to regulate *CES1* expression, promoting recurrence and metastasis of head and neck squamous carcinoma.

Duvvuri et al. [52] discovered that in head and neck squamous carcinoma, TMEM16A is overexpressed, correlating with a decrease in overall patient survival. This study indicates that TMEM16A-induced cancer cell proliferation and tumor growth are accompanied by the activation of the ERK1/2 signaling pathway. The use of drugs to inhibit MEK/ERK and genetic inactivation of ERK1/2 eliminated the growth effects of TMEM16A, suggesting that MAPK activation plays a crucial role in TMEM16A-mediated proliferation. Xie et al. [53] showed that the MEK inhibitor trametinib, through inhibition of the MAPK-ERK signaling pathway and EGFR, along with a reduction in the pro-survival protein MYC and an increase in the expression of MYC-targeted cyclin-dependent kinase inhibitor p27kip1 and the pro-apoptotic protein BIM, combats head and neck squamous carcinoma. In our transcriptomic study, GO enrichment analysis of the NC and SH groups revealed the regulation of pathways, such as cell migration, cell adhesion, and positive regulation of cell migration. KEGG enrichment analysis indicated that these regulations were related to pathways including the MAPK signaling pathway. Western blot experiments showed that the levels of p-MEK/MEK, p-ERK/ERK, and CES1 decreased with the knockdown of *CHRNA5*. The ERK/ERK pathway inhibitor, PD98059, further reduced these levels in the SH group, whereas nicotine reversed this trend. PD98059 was also able to inhibit the nicotine-induced increase in p-MEK/MEK, p-ERK/ERK, and CES1 levels, indicating that *CHRNA5* regulates *CES1* expression through the MEK/ERK pathway (Fig. 12).

Ahmed et al. [54] indicated that compared to adjacent normal tissues, the mRNA and protein levels of Ki-67 are significantly increased in patients with head and neck squamous carcinoma, suggesting that overexpression of Ki-67 may be associated with the occurrence and progression of head and neck squamous carcinoma. Dumitru [55] studied 50 cases with head and neck squamous carcinoma patients and found that high expression of E-cadherin was mainly associated with tumors of lower differentiation (*p* = 0.0305), and there was a significant correlation between the expression of Ki-67 in tumor cells and tumor grading (*p* = 0.0245). A strong correlation was observed between low E-cadherin expression, increased Ki-67 proliferation rate, and progression to T2-T3 tumor stages (*p* = 0.0242), with high Ki-67 expression associated with low E-cadherin expression, indicating a poorer prognosis. Lee [56] investigated the therapeutic potential of epigenetic reprogramming of EMT in promoting ferroptosis in head and neck squamous carcinoma cells and found that high E-cadherin expression is associated with reduced susceptibility to ferroptosis. Groeger [57] studied immune evasion in oral squamous carcinoma using pan-ck staining to provide evidence of the epithelial origin of tissues. Hinić [58] utilized gene expression and DNA methylation data from TCGA-HNSC to compare HPV-positive and HPV-negative groups, and differential expression analysis revealed 1854 differentially expressed genes, including PCNA. Wei et al. [59] found that the proteasome inhibitors MG-262 and bepridil might interfere with the progression of head and neck squamous carcinoma by targeting PCNA. We found that the tumor volume in nude mice significantly decreased with knockdown of *CHRNA5* (*p* < 0.0001), and nicotine treatment increased the tumor volume (*p* < 0.001). Tumor western blot experiments and qRT-PCR validation confirmed that p-MEK/MEK, p-ERK/ERK, and CES1 levels decreased with the knockdown of CHRNA5, whereas nicotine reversed this trend in the SH group, indicating that *CHRNA5* regulates *CES1* expression through the MEK/ERK pathway. Immunohistochemical analysis of CHRNA5, CES1, Pan-ck, PCNA, Ki-67, and E-cadherin in tumor tissues and human laryngeal and hypopharyngeal cancer specimens showed that *CHRNA5* regulates *CES1* expression via the MEK/ERK pathway, affecting the recurrence and metastasis of head and neck squamous carcinoma (Fig. 12).

## Conclusions

Nicotine enhances the proliferation, migration, and invasion abilities of head and neck tumor cells by promoting the expression of CHRNA5. Knockdown of CHRNA5 reduced cell proliferation, migration, and invasion capabilities, whereas nicotine exposure reversed this trend. CHRNA5 and CES1 can form a stable protein docking model, as well as form an immunoprecipitation with a high degree of colocalization distribution. Transcriptome enrichment analysis revealed that knockdown of CHRNA5 is related to the MAPK signaling pathway, among others. Cell-level western blot experiments confirm that CHRNA5 regulates CES1 expression through the MEK/ERK pathway, affecting the recurrence and metastasis of head and neck squamous carcinoma. Tissue-level western blot experiments and specimen immunohistochemistry further indicate that CHRNA5 influences the recurrence and metastasis of head and neck squamous carcinoma through the MEK/ERK pathway by regulating CES1 expression.

## Supplementary information

FIGURE S1

Supplemental Figure legend

Original Data

## Data Availability

All data generated or analyzed during this study are included in this published article.
