# Correction: Nicotine-induced *CHRNA5* activation modulates *CES1* expression, impacting head and neck squamous cell carcinoma recurrence and metastasis via MEK/ERK pathway

**DOI:** 10.1038/s41419-024-07261-w

**Published:** 2024-12-20

**Authors:** Chen Feng, Wei Mao, Chenyang Yuan, Pin Dong, Yuying Liu

**Affiliations:** 1 Department of Otolaryngology, Head and Neck Surgery, Shanghai General Hospital, Shanghai Jiao Tong University School of Medicine, Shanghai, China; 2 Department of Otolaryngology, Head and Neck Surgery, Qilu Hospital, Shandong University Cheeloo College of Medicine, NHC Key Laboratory of Otorhinolaryngology (Shandong University), Jinan, China; 3 Department of Otorhinolaryngology, Head and Neck surgery, The First Hospital affiliated to Harbin Medical University, Harbin, Heilongjiang China

**Keywords:** Head and neck cancer, Diagnostic markers

Correction to: *Cell Death and Disease* 10.1038/s41419-024-07178-4, published online 29 October 2024

Unfortunately, due to an oversight, the version of Figure 3A was the pre-submission version. We sincerely apologize for this mistake and any inconvenience it may have caused.

Wrong image:
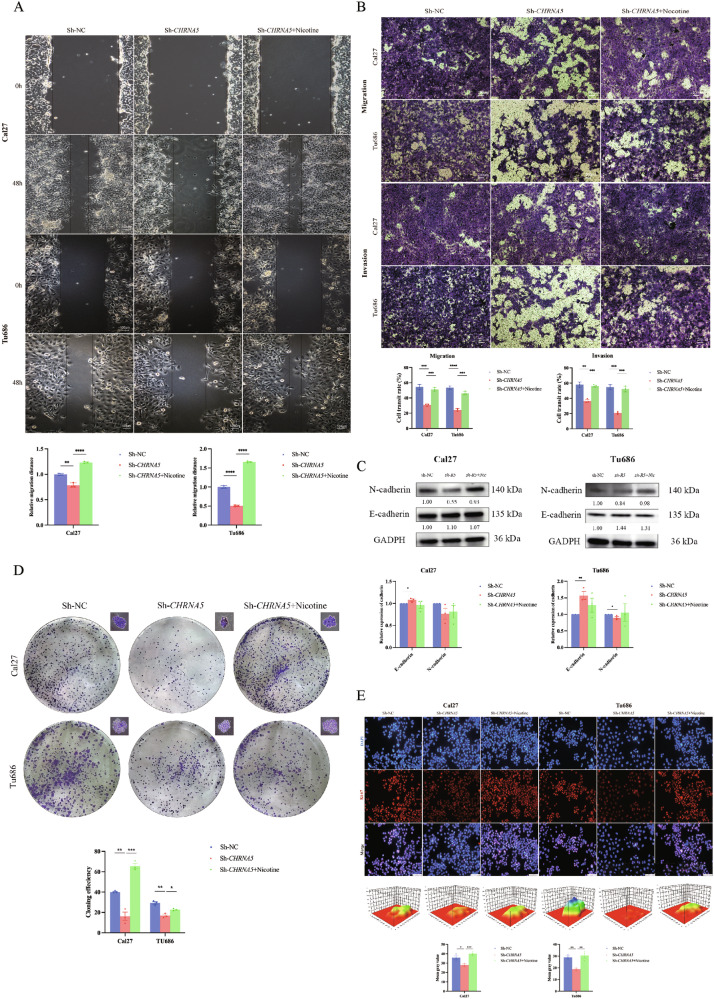


Corrected image:
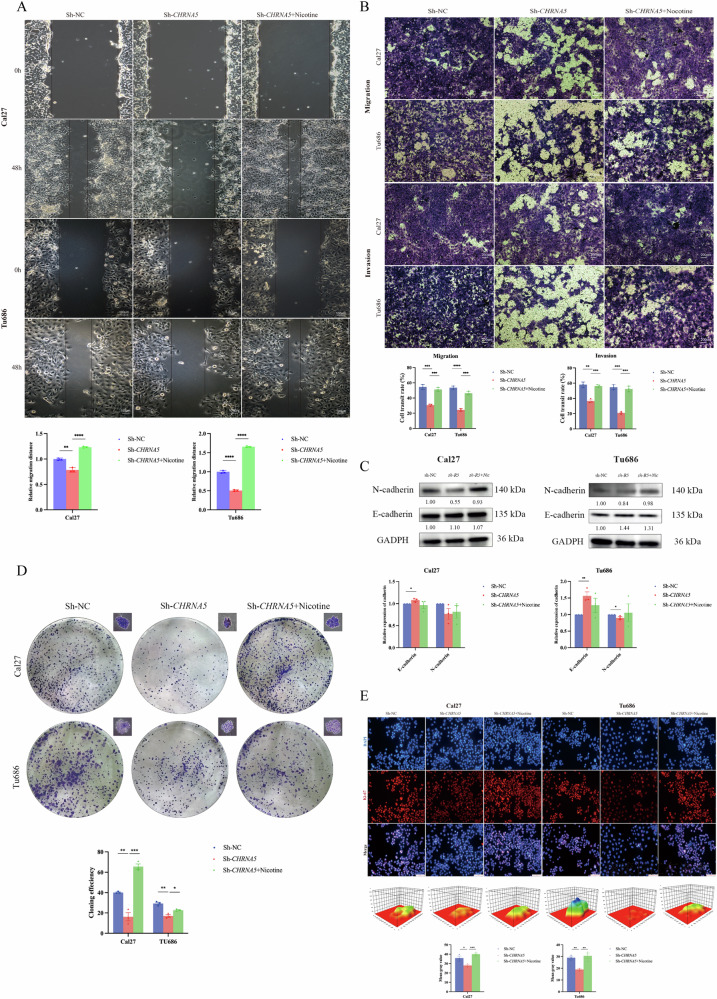


The original article has been corrected.

