# Supplementary figures and images for: Nicotine-induced CHRNA5 activation modulates CES1 expression, impacting head and neck squamous cell carcinoma recurrence and metastasis via MEK/ERK pathway
Source: Cell Death Dis. 2024 Oct 29;15(10):785. doi: 10.1038/s41419-024-07178-4 (PMC11522702; doi:10.1038/s41419-024-07178-4)

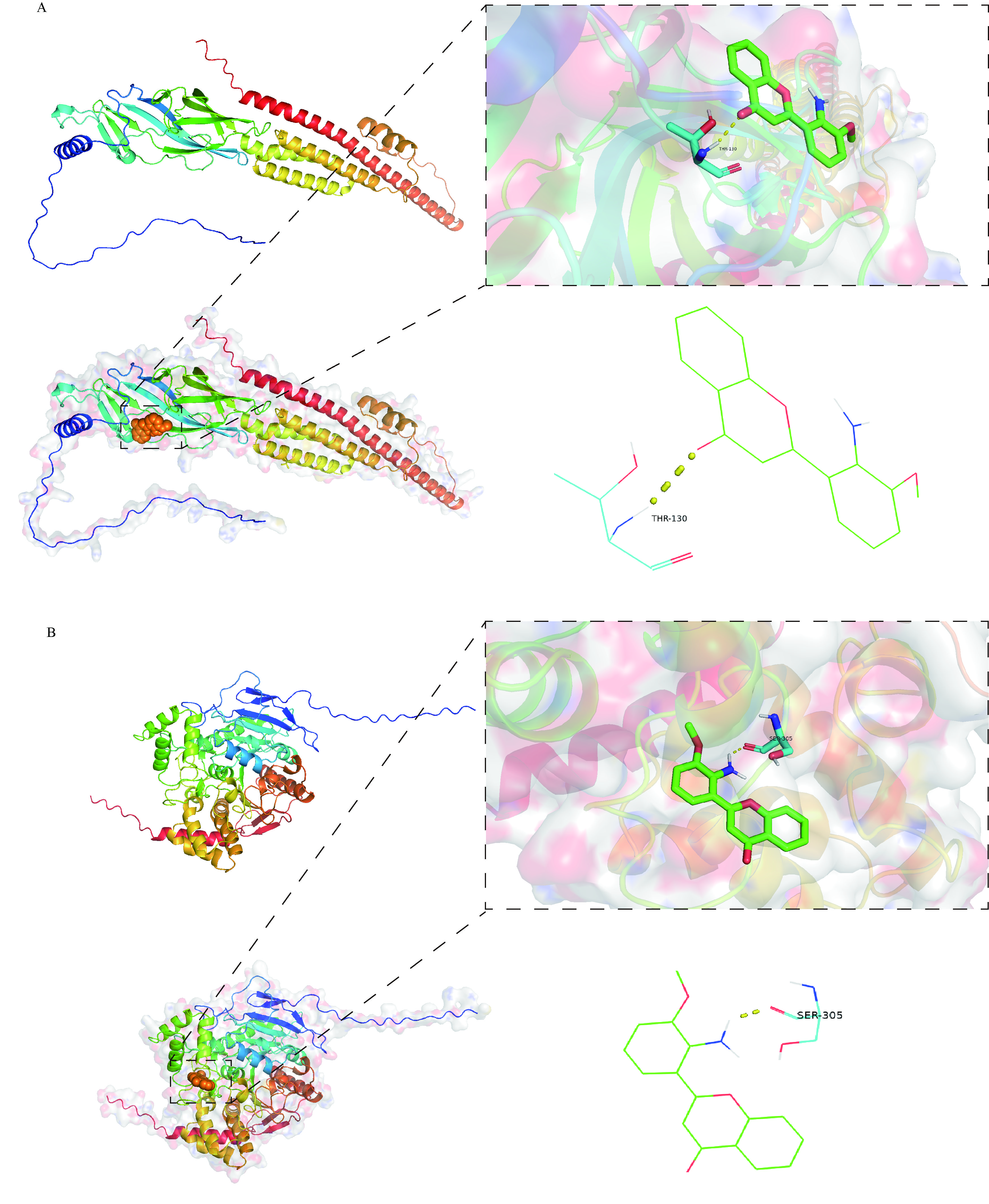

Supplement: Supplementary file 1 — FIGURE S1 [file 41419_2024_7178_MOESM1_ESM.jpg]

GAPDH-02

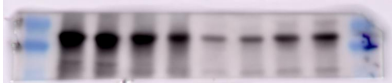

CHRNA5-02

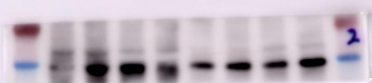

FULL-02

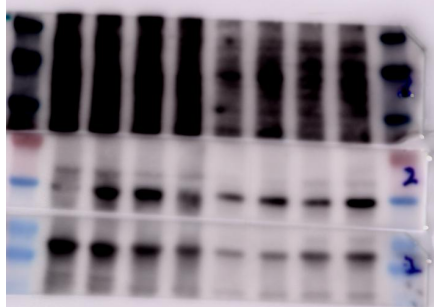

Supplement: Supplementary file 3 — Original Data [file 41419_2024_7178_MOESM3_ESM.zip › Supplemental Material/Figure 2B.pdf]

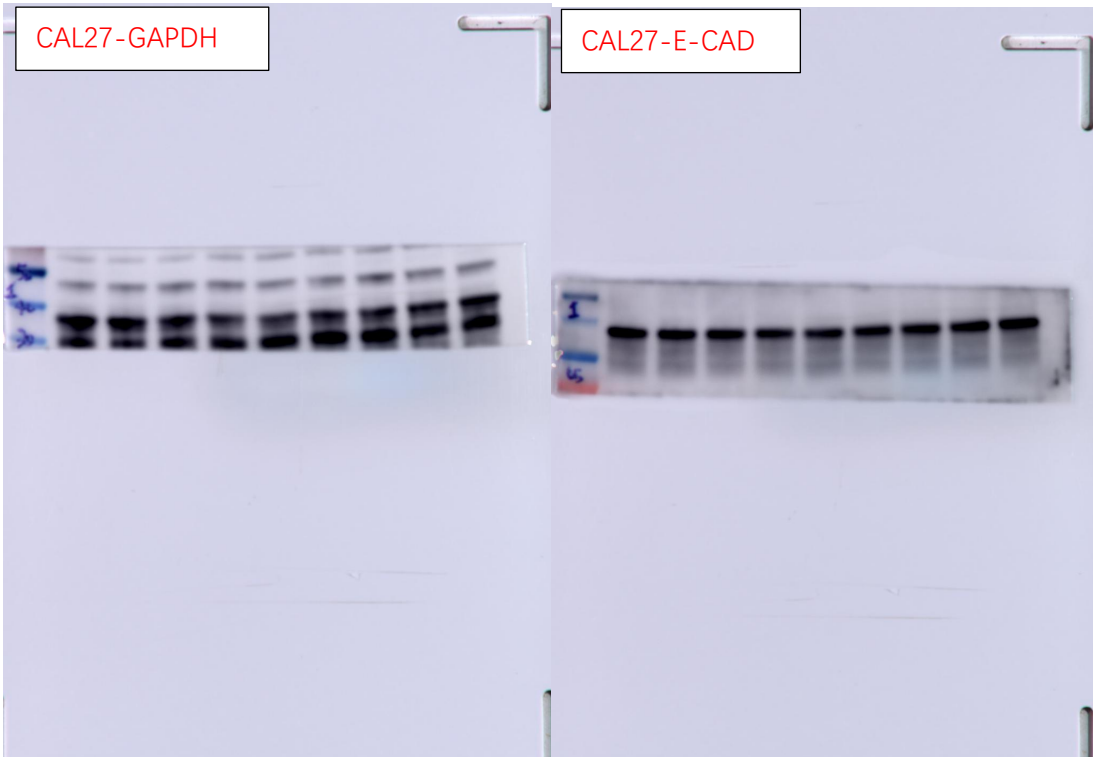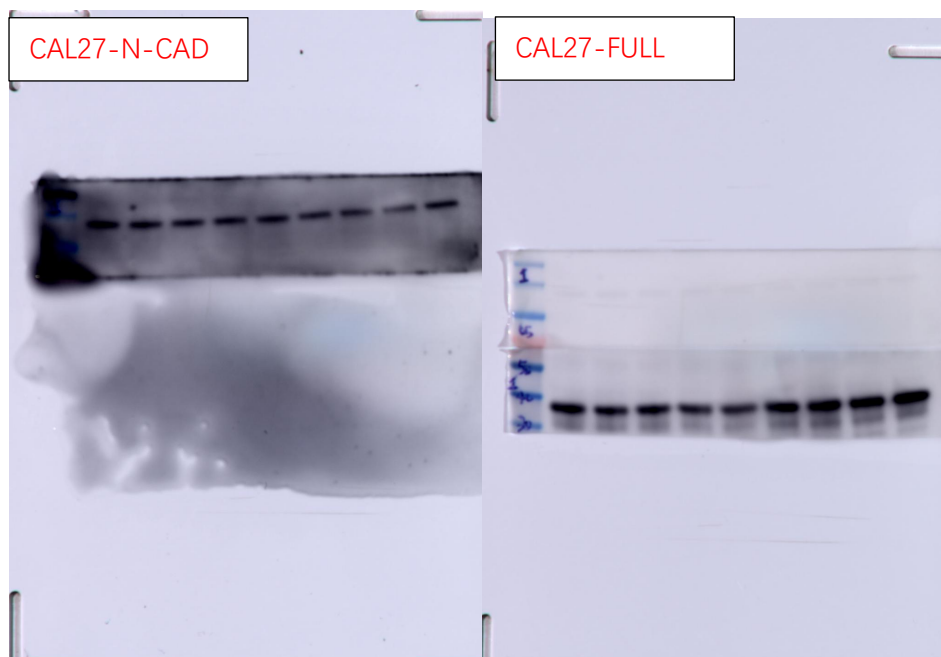

TU686-GAPDH

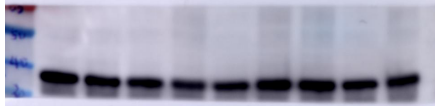

TU686-E-CAD

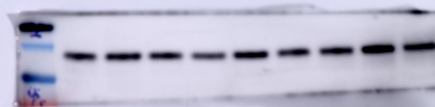

TU686-N-CAD

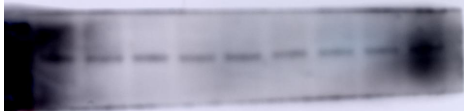

TU686-FULL

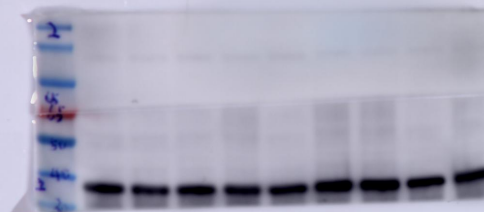

Supplement: Supplementary file 3 — Original Data [file 41419_2024_7178_MOESM3_ESM.zip › Supplemental Material/Figure 3C.pdf]

GAPDH

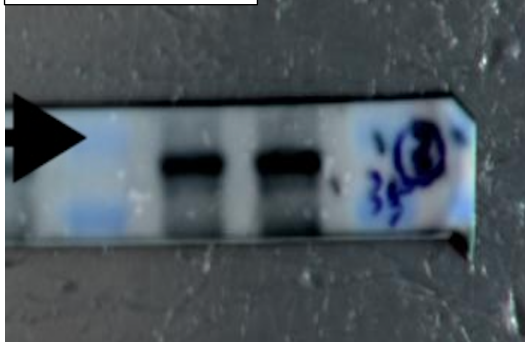

N-CAD

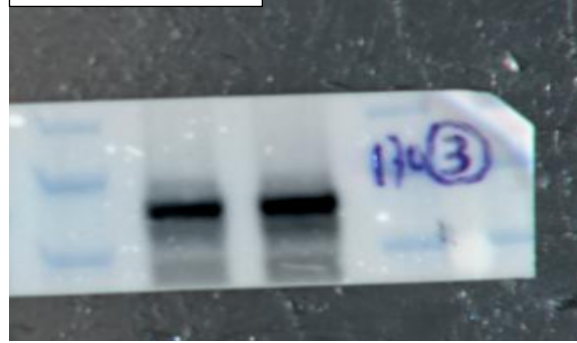

N-CAD

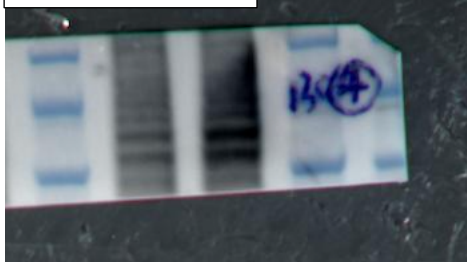

FULL

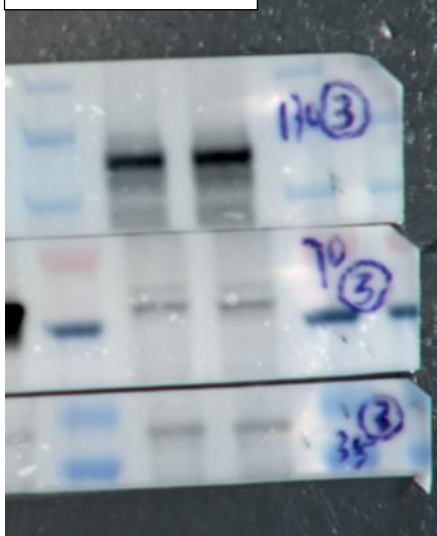

FULL

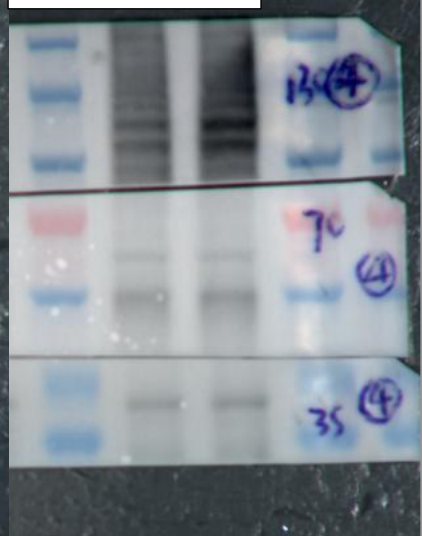

E-CAD

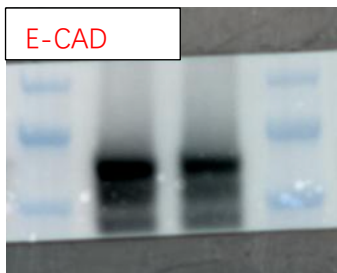

FULL

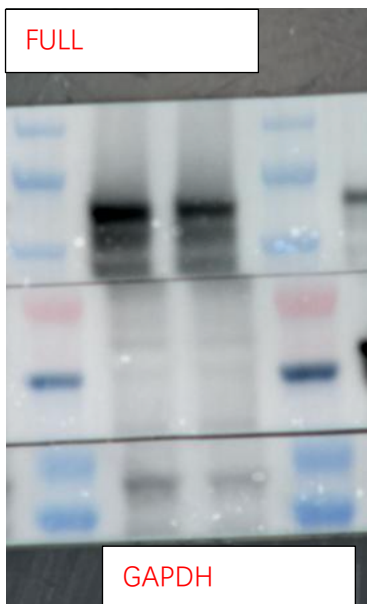

GAPDH

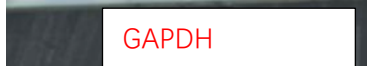

Supplement: Supplementary file 3 — Original Data [file 41419_2024_7178_MOESM3_ESM.zip › Supplemental Material/Figure 4C.pdf]

CAL27-GAPDH

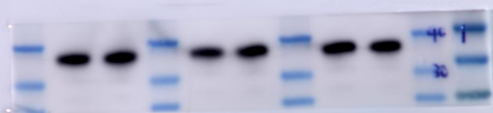

CAL27-CHRNA5

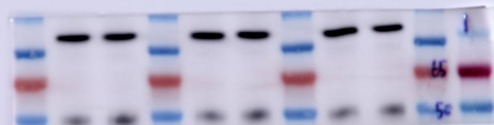

CAL27-FULL

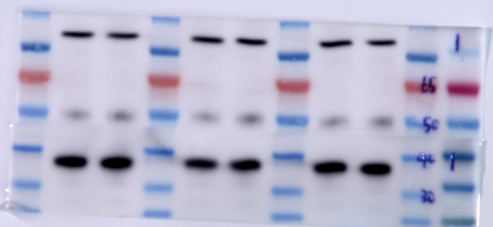

TU686-FULL

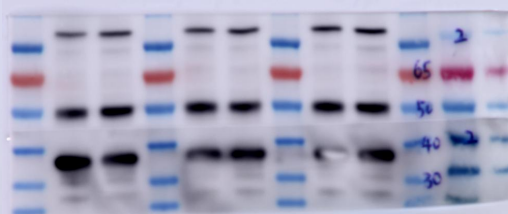

TU686-GAPDH

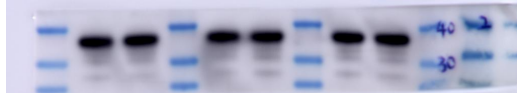

TU686-CHRNA5

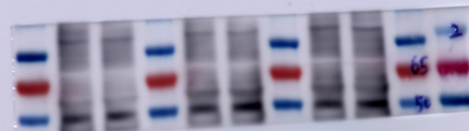

Supplement: Supplementary file 3 — Original Data [file 41419_2024_7178_MOESM3_ESM.zip › Supplemental Material/Figure 7B.pdf]

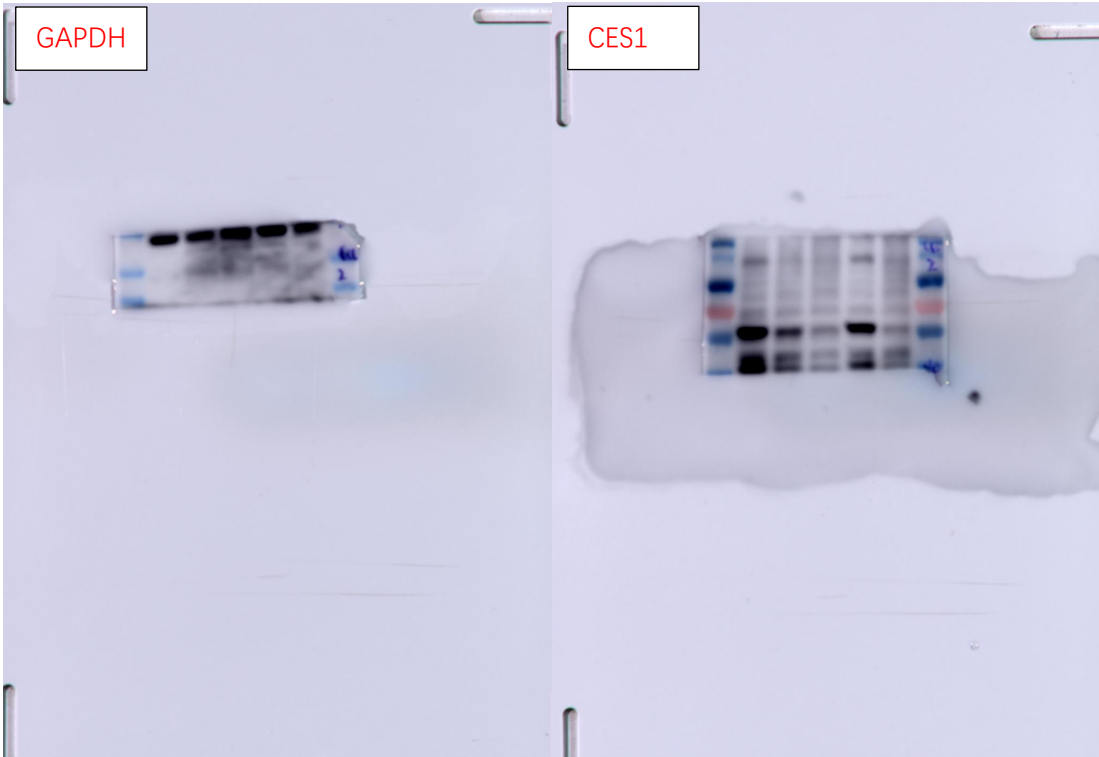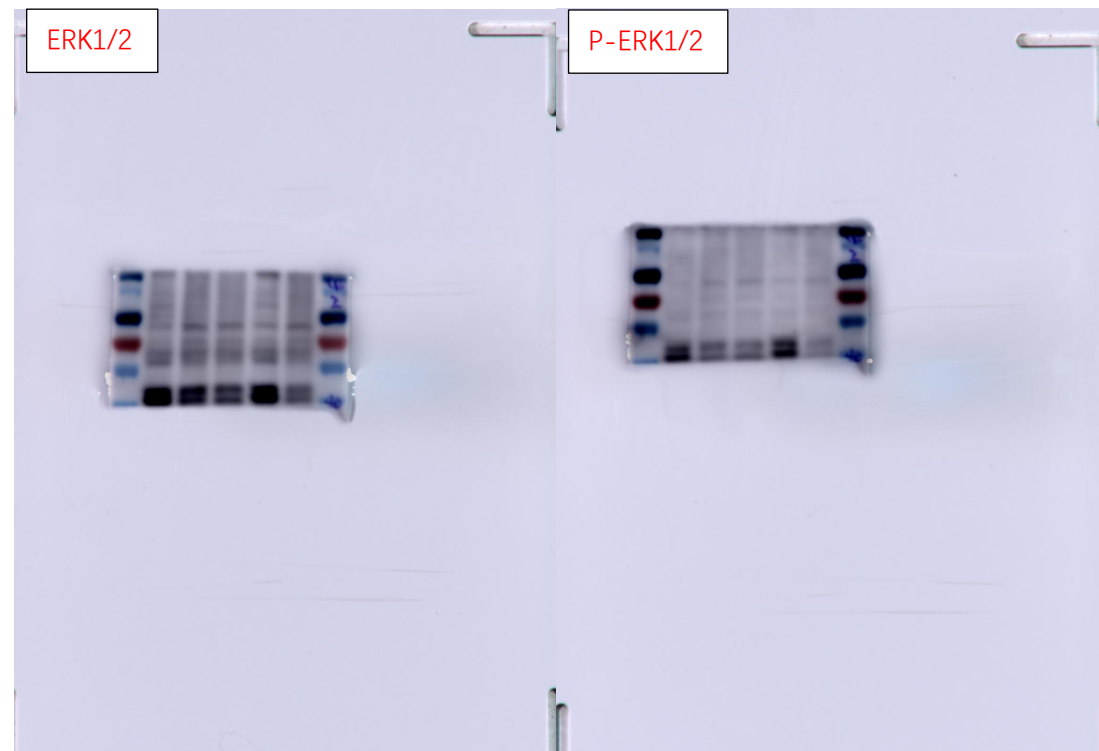

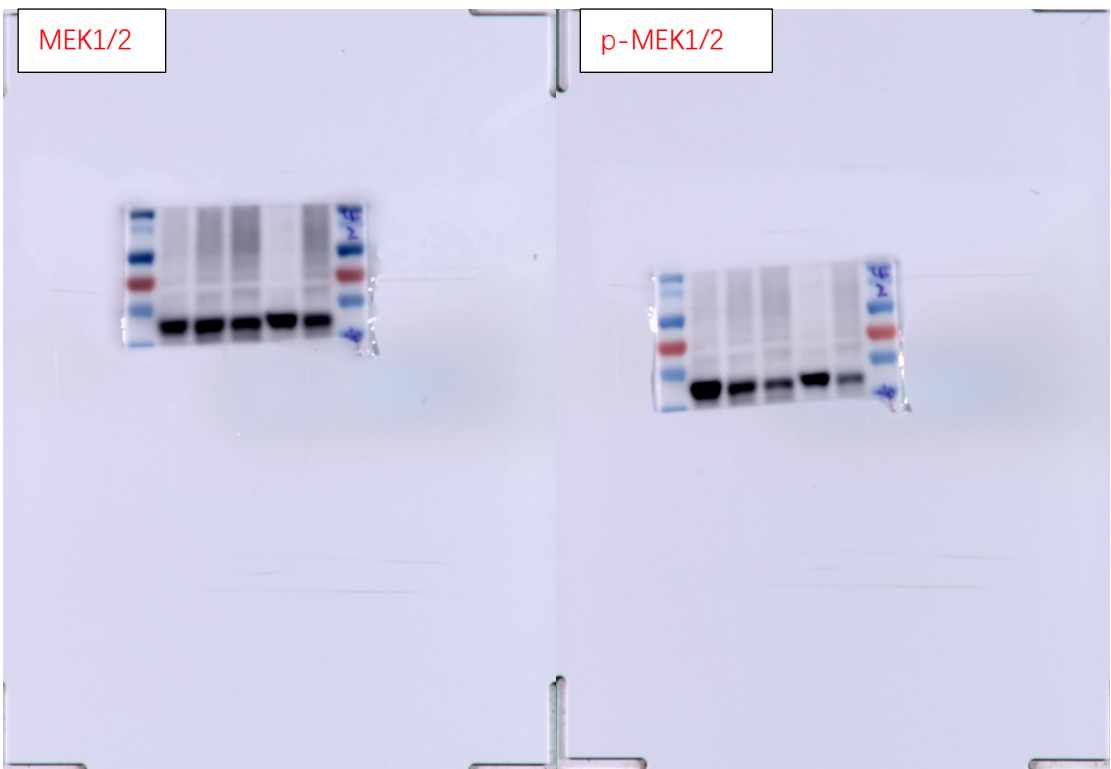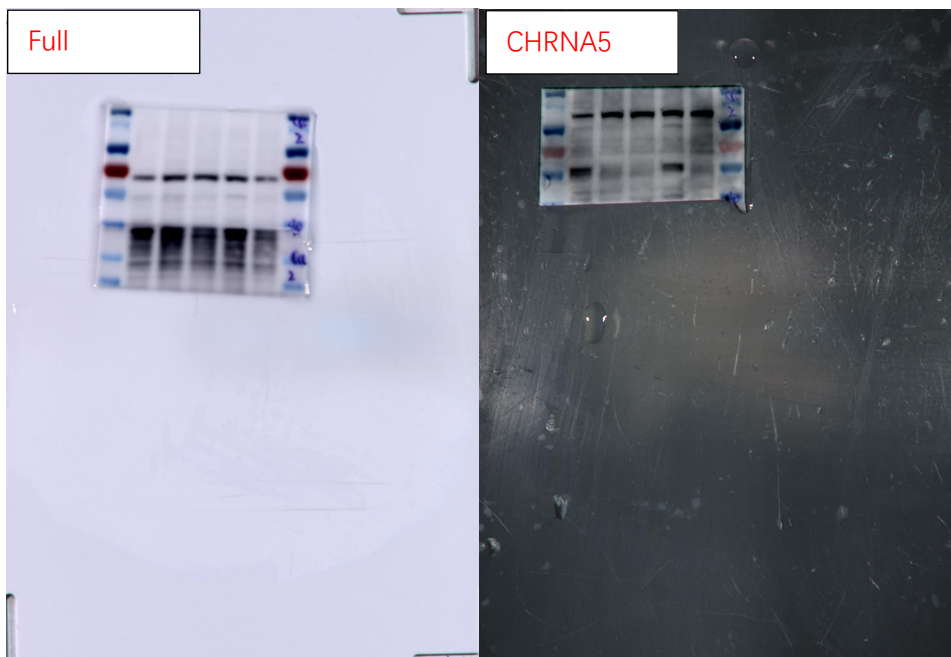

Supplement: Supplementary file 3 — Original Data [file 41419_2024_7178_MOESM3_ESM.zip › Supplemental Material/Figure 8A.pdf]

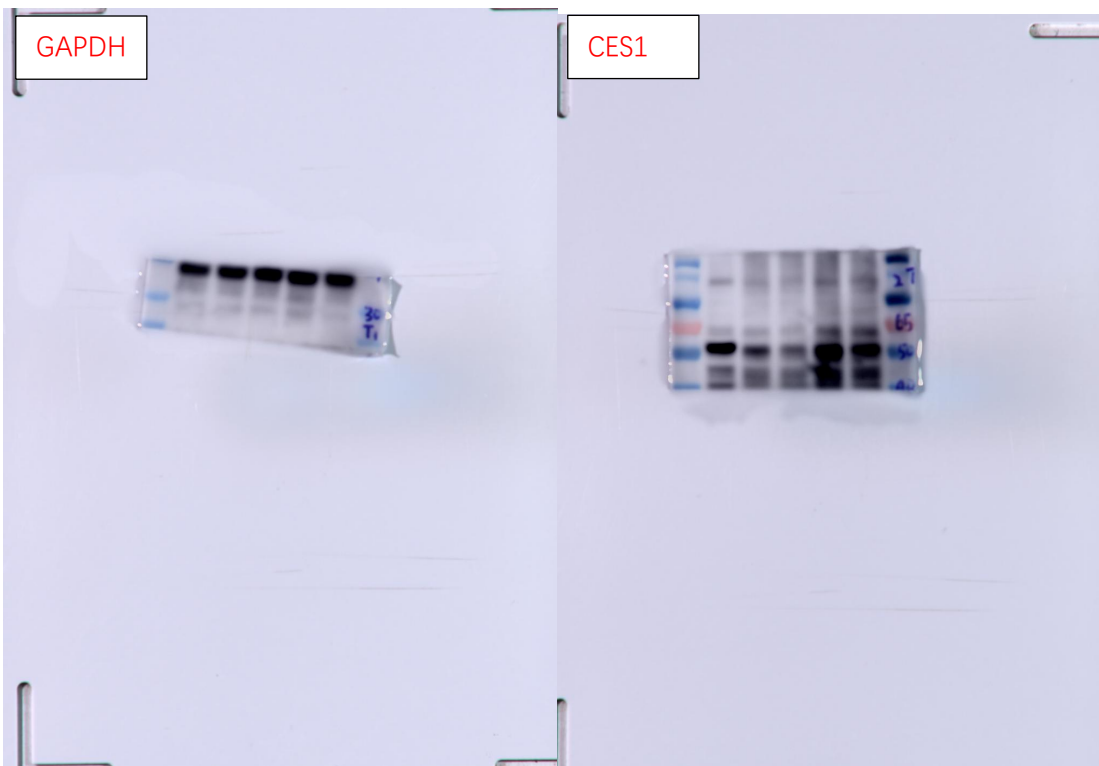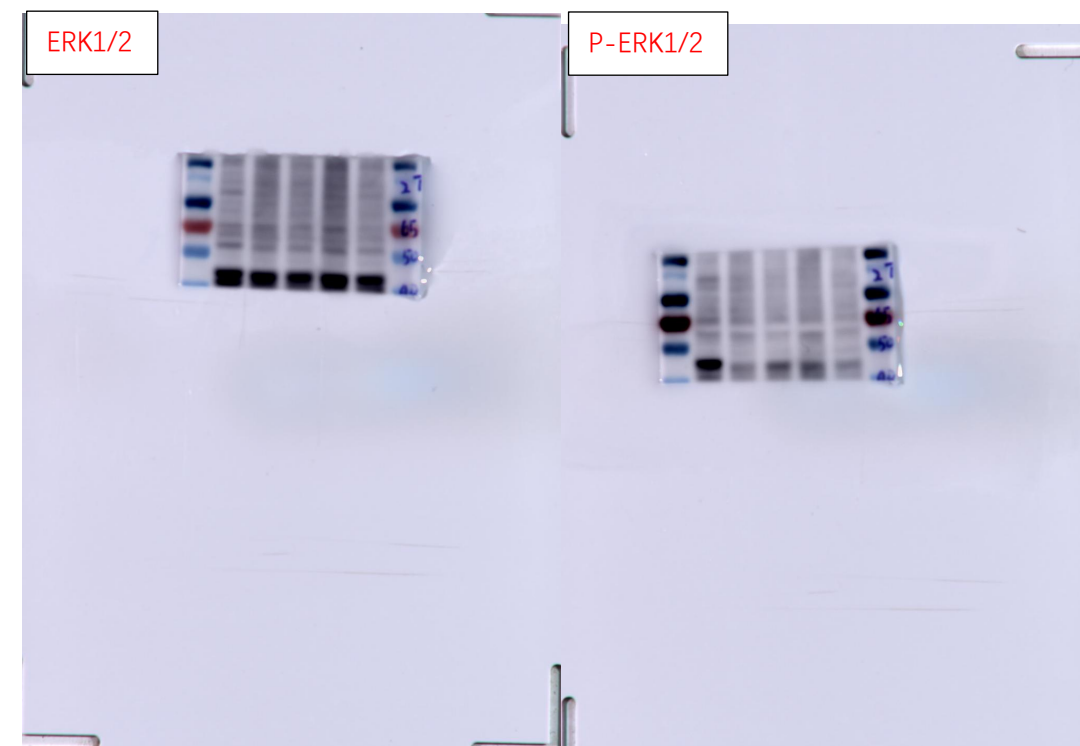

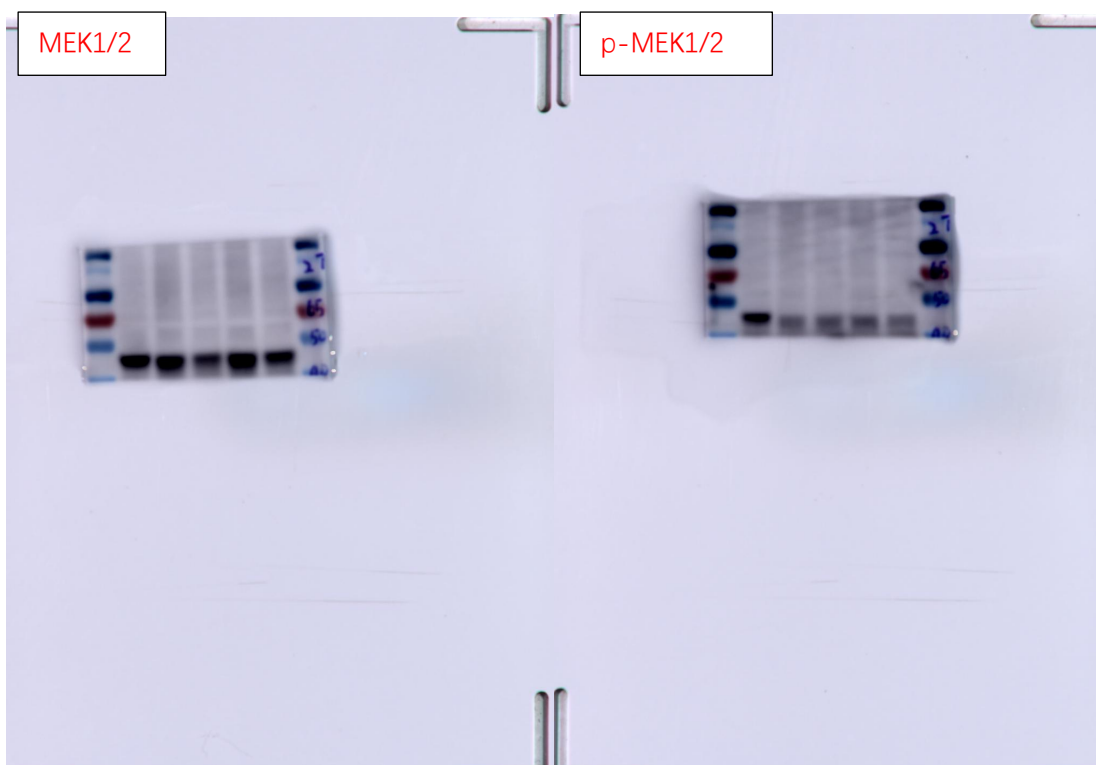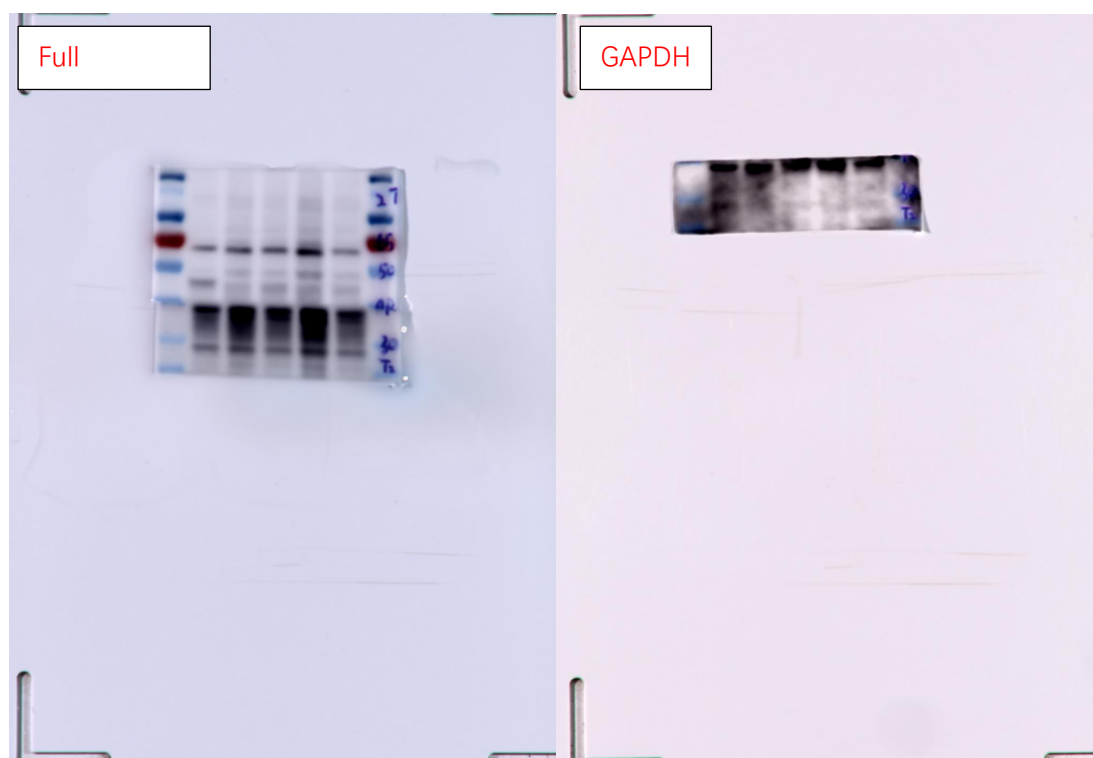

CHRNA5

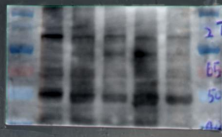

Supplement: Supplementary file 3 — Original Data [file 41419_2024_7178_MOESM3_ESM.zip › Supplemental Material/Figure 8B.pdf]

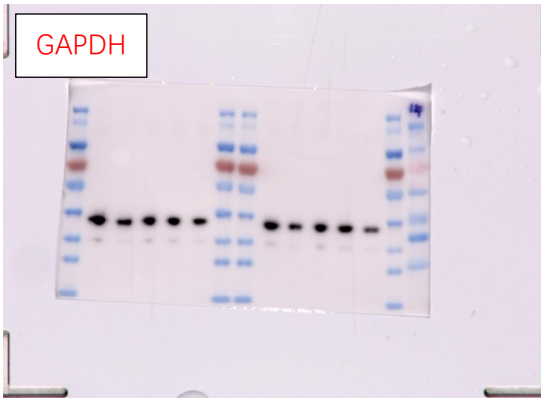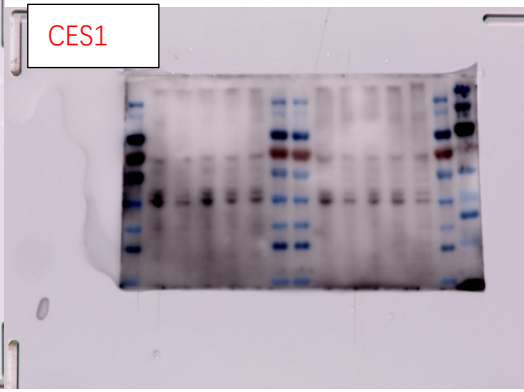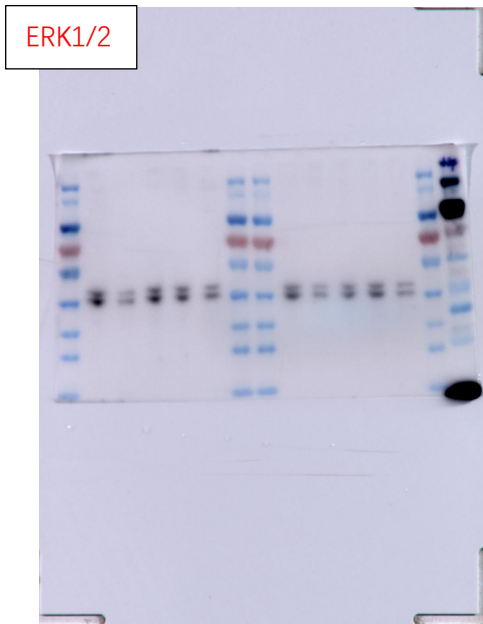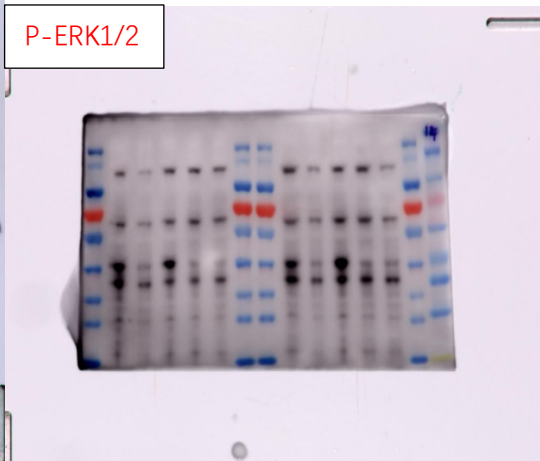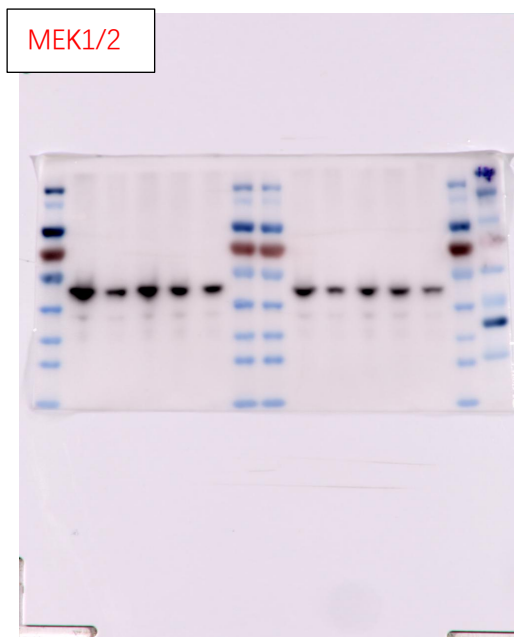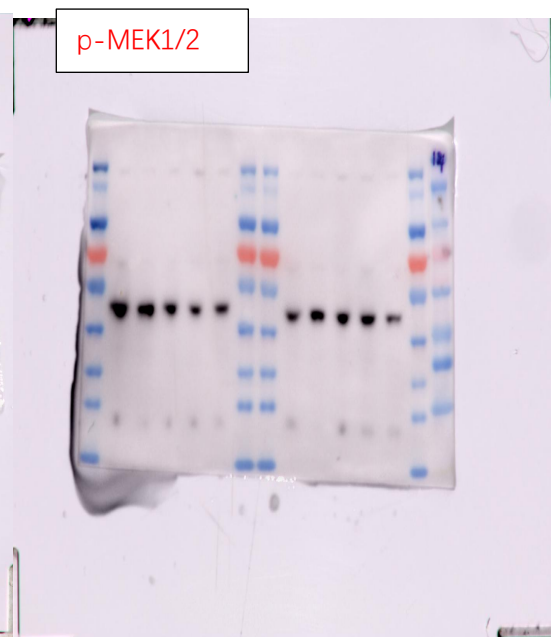

CHRNA5

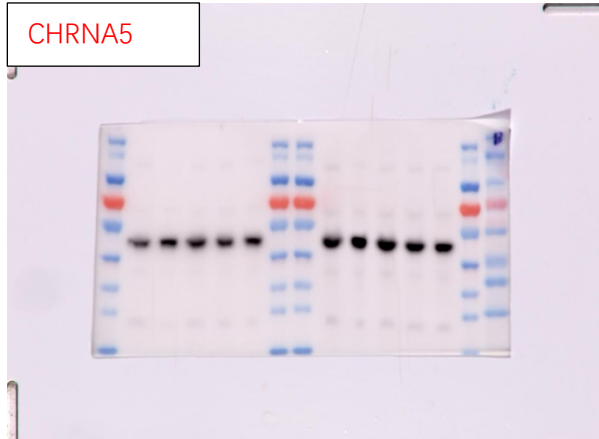

Supplement: Supplementary file 3 — Original Data [file 41419_2024_7178_MOESM3_ESM.zip › Supplemental Material/Figure 8D.pdf]

GAPDH

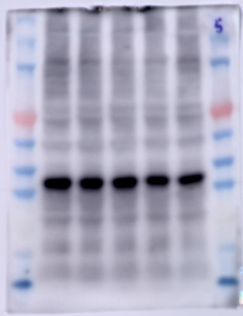

CES1

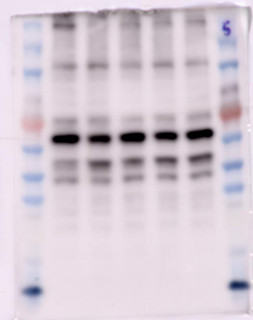

ERK1/2

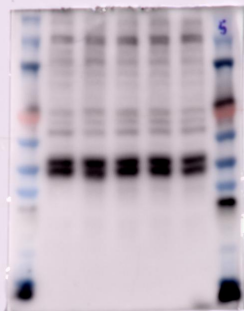

P-ERK1/2

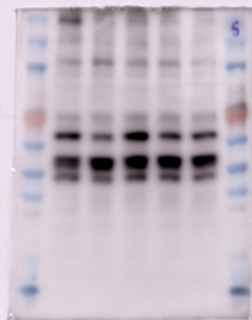

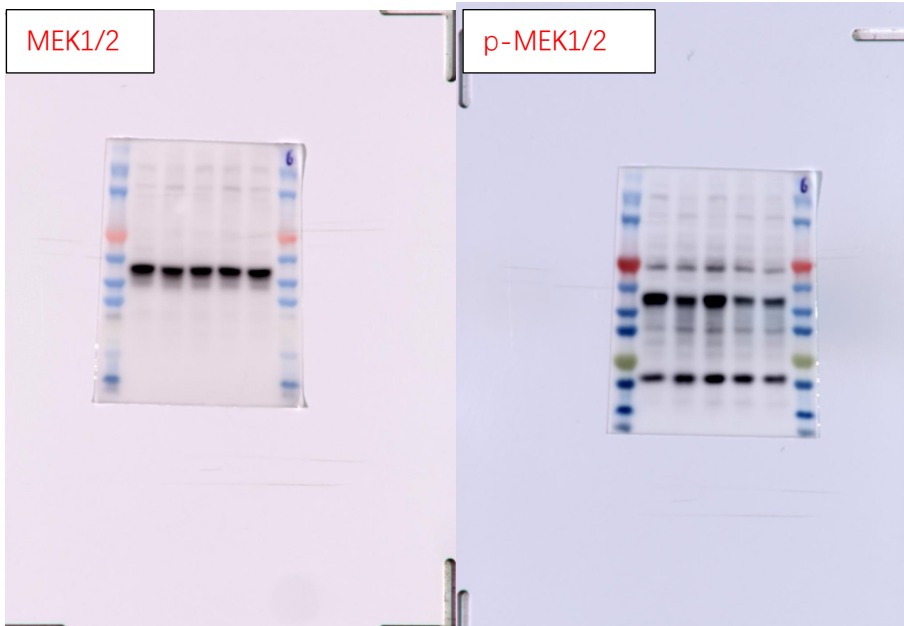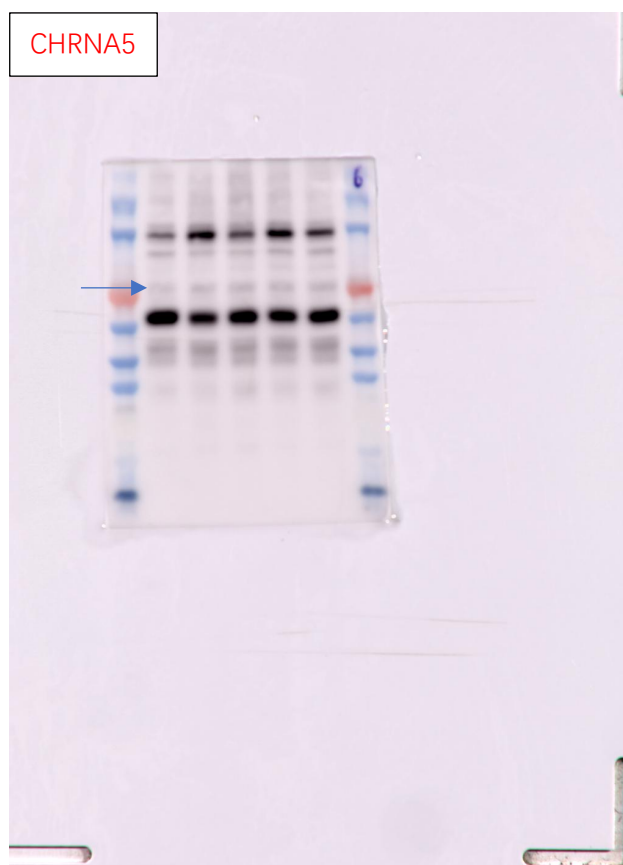

Supplement: Supplementary file 3 — Original Data [file 41419_2024_7178_MOESM3_ESM.zip › Supplemental Material/Figure 8E.pdf]

GAPDH

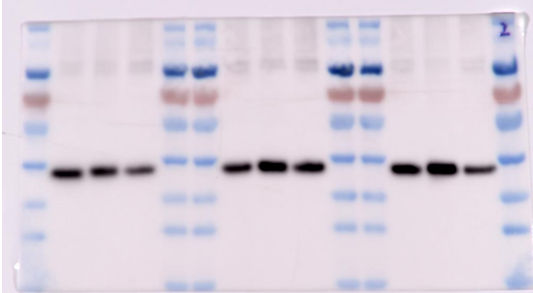

CES1

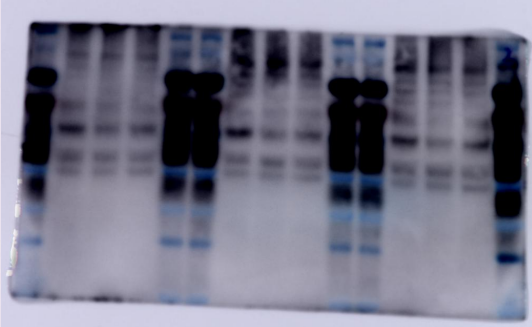

ERK1/2

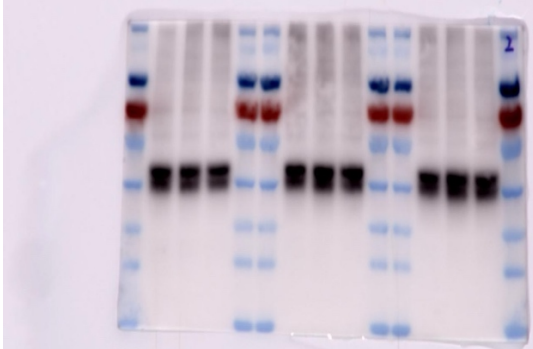

P-ERK1/2

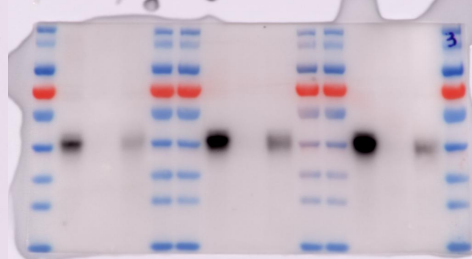

MEK1/2

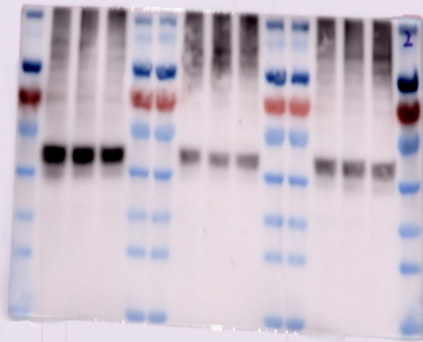

p-MEK1/2

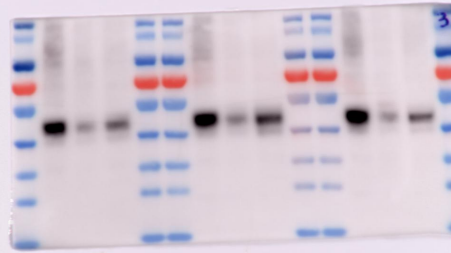

Supplement: Supplementary file 3 — Original Data [file 41419_2024_7178_MOESM3_ESM.zip › Supplemental Material/Figure 9G.pdf]
