# Supplementary material for: Nicotine-induced CHRNA5 activation modulates CES1 expression, impacting head and neck squamous cell carcinoma recurrence and metastasis via MEK/ERK pathway
Source: Cell Death Dis. 2024 Oct 29;15(10):785. doi: 10.1038/s41419-024-07178-4 (PMC11522702; doi:10.1038/s41419-024-07178-4)
Supplement: Supplementary file 2 — Supplemental Figure legend [file 41419_2024_7178_MOESM2_ESM.docx]

**Fig. S1** Docking diagram of the small molecule PD98059 with the protein structures of CHRNA5(A) and CES1(B)
