# Supplementary material for: Nicotine-induced CHRNA5 activation modulates CES1 expression, impacting head and neck squamous cell carcinoma recurrence and metastasis via MEK/ERK pathway
Source: Cell Death Dis. 2024 Oct 29;15(10):785. doi: 10.1038/s41419-024-07178-4 (PMC11522702; doi:10.1038/s41419-024-07178-4)

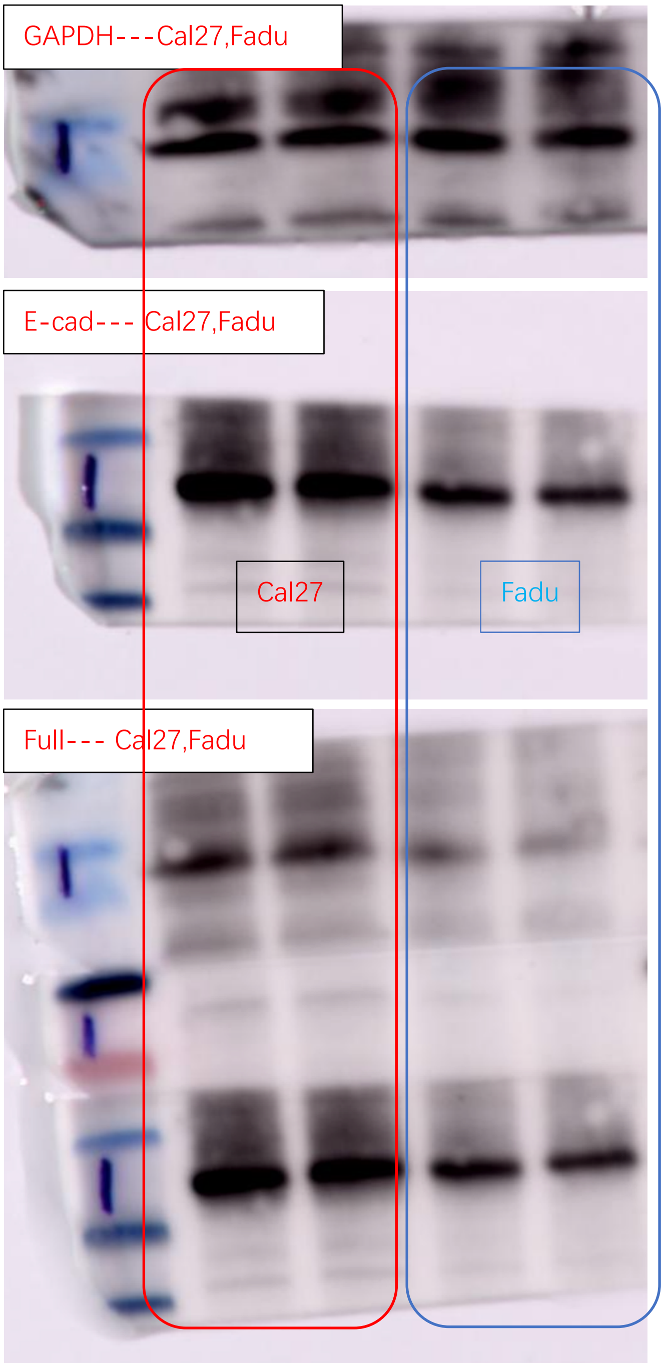
GAPDH---Cal27,Fadu
E-cad--- Cal27,Fadu
Cal27
Fadu
Full--- Cal27,Fadu

Full-E-CAD--- HN6

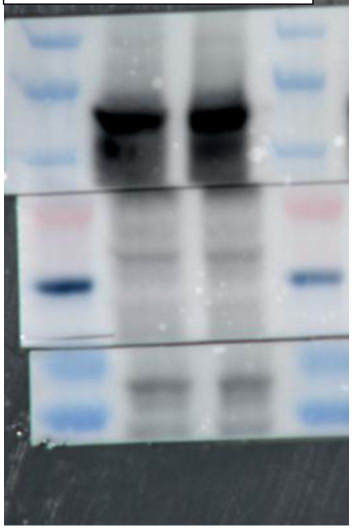

GAP--- Tu686

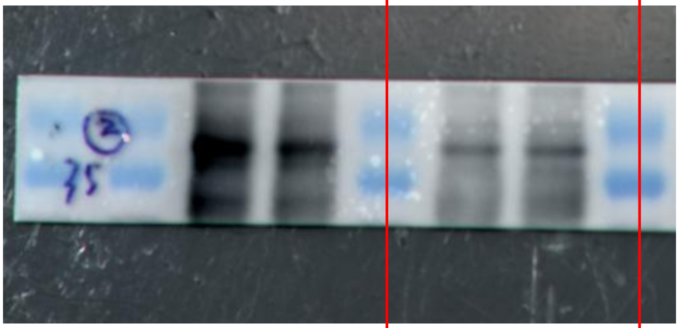

E-cad--- Tu686

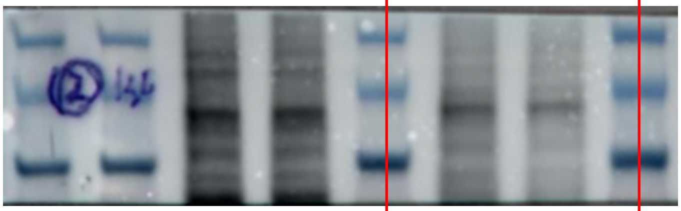

Full--- Tu686

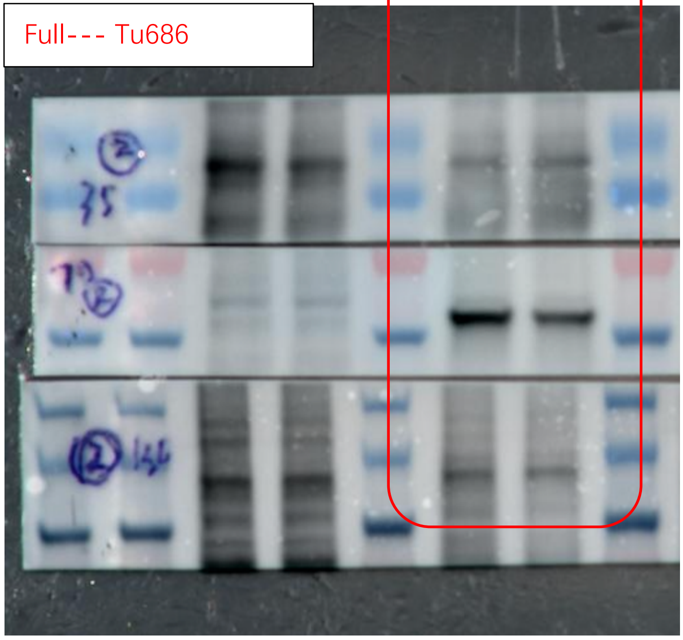

GAPDH---Cal27

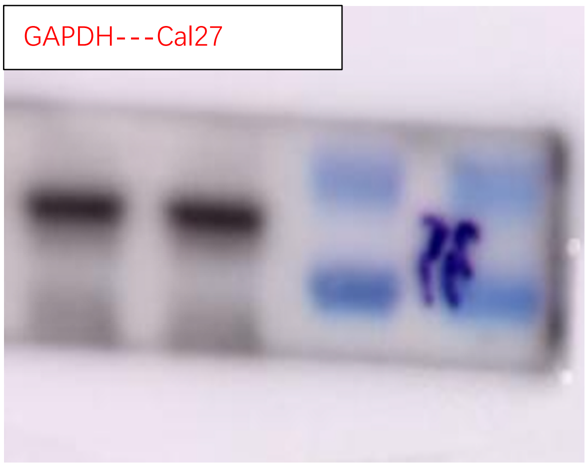

N-cad---Cal27

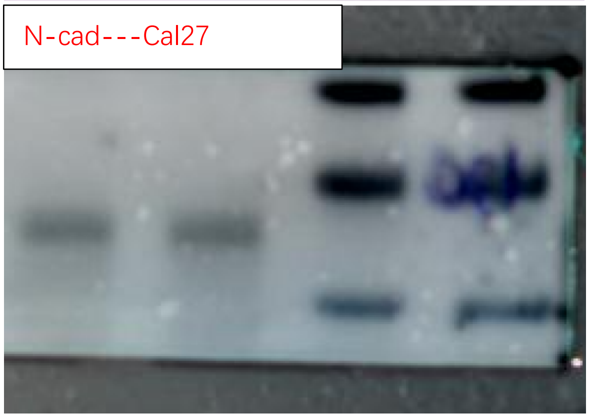

Full---Cal27(ps.E-CAD)

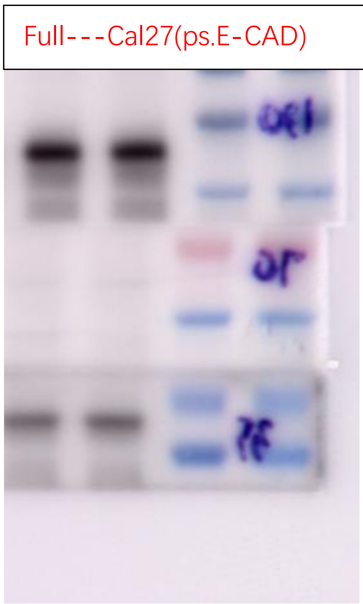

GAPDH---Fadu

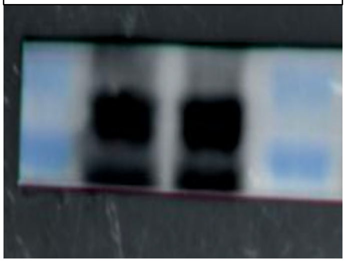

N-cad---Fadu

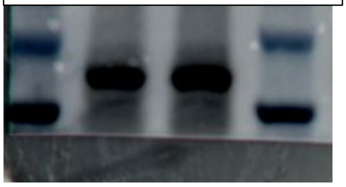

Full---Fadu

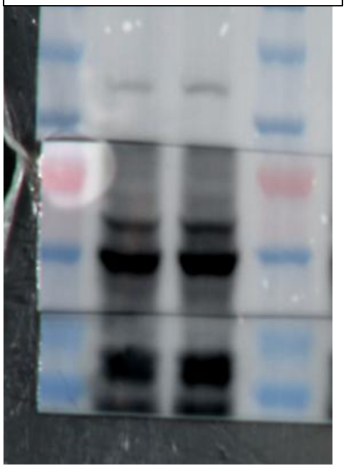

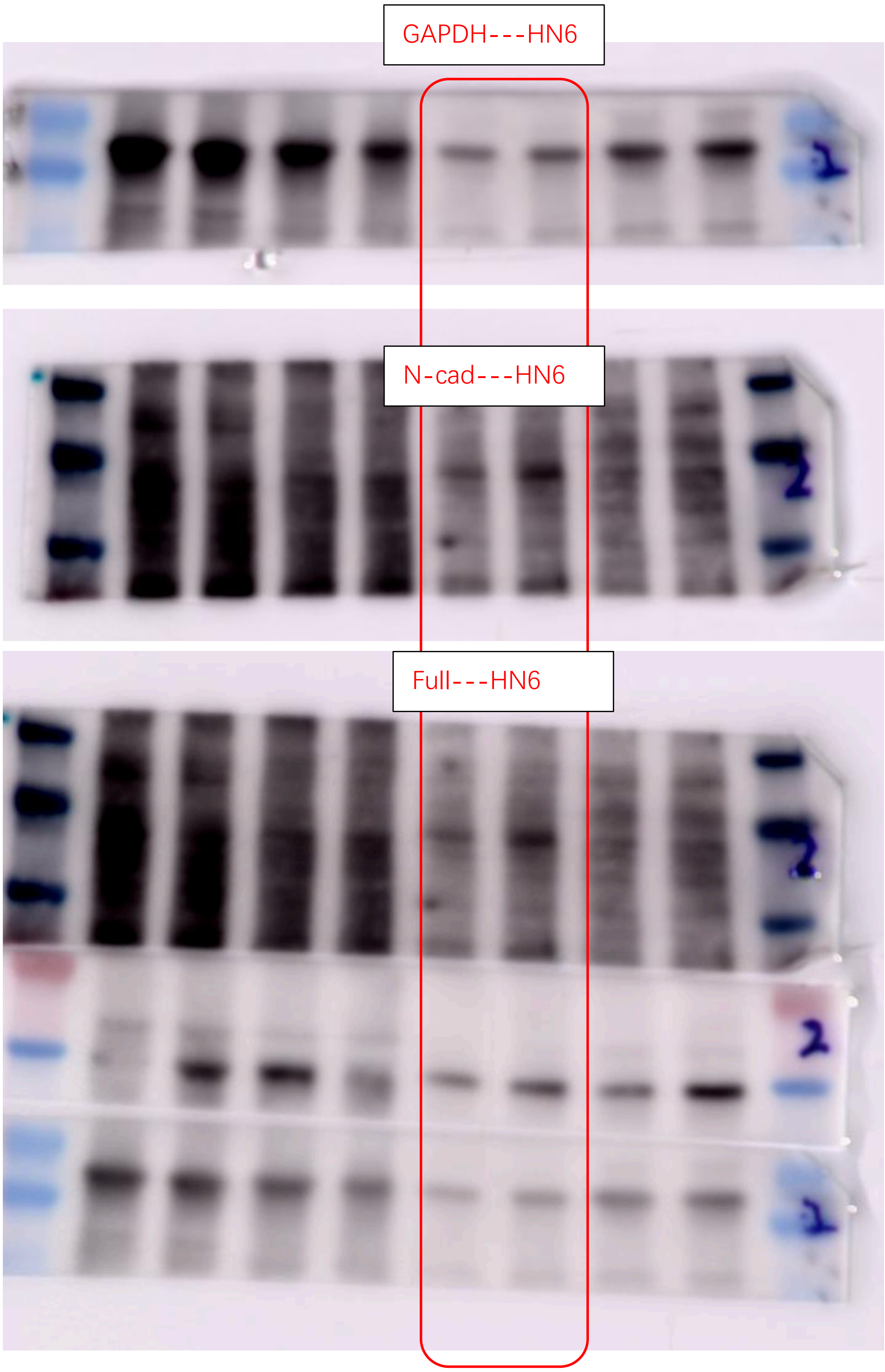
GAPDH---HN6
N-cad---HN6
Full---HN6

GAPDH---Tu686

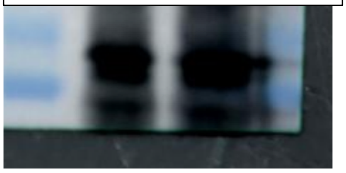

N-cad---Tu686

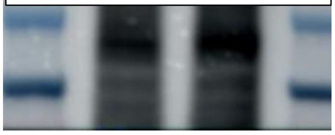

Full---Tu686

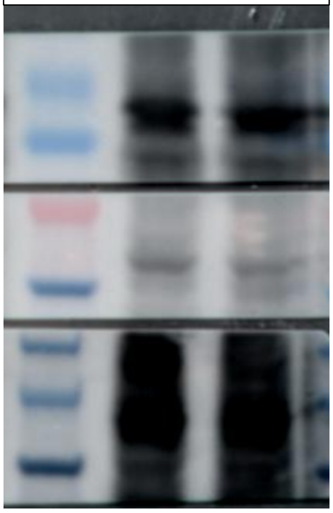

Supplement: Supplementary file 3 — Original Data [file 41419_2024_7178_MOESM3_ESM.zip › Supplemental Material/Figure 1B.pdf]
