# Supplementary material for: Nicotine-induced CHRNA5 activation modulates CES1 expression, impacting head and neck squamous cell carcinoma recurrence and metastasis via MEK/ERK pathway
Source: Cell Death Dis. 2024 Oct 29;15(10):785. doi: 10.1038/s41419-024-07178-4 (PMC11522702; doi:10.1038/s41419-024-07178-4)

CAL27-FLAG

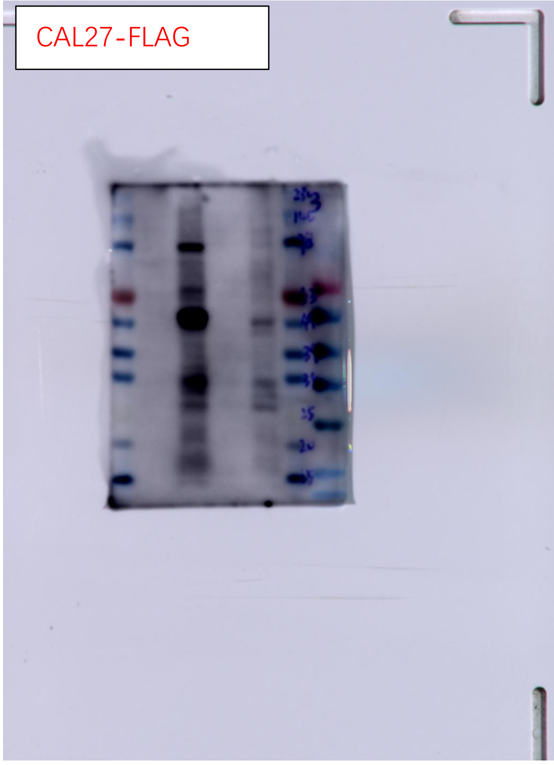

CAL27-CES1

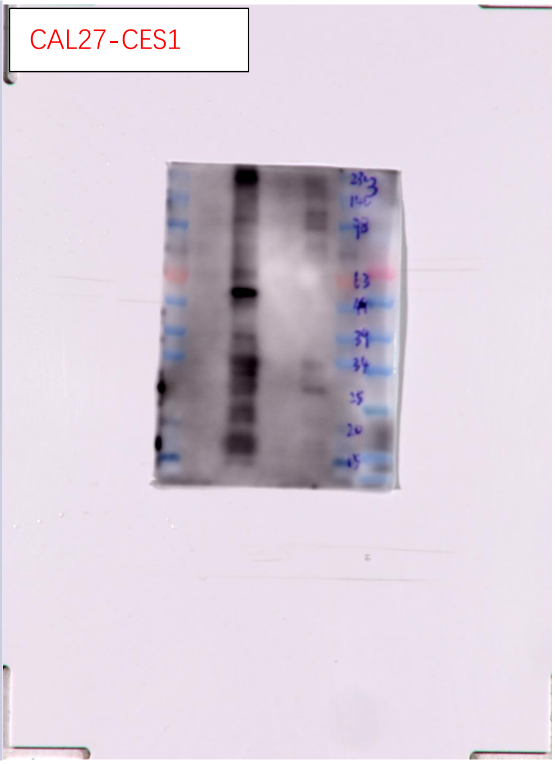

INPUT-CAL27-GAP

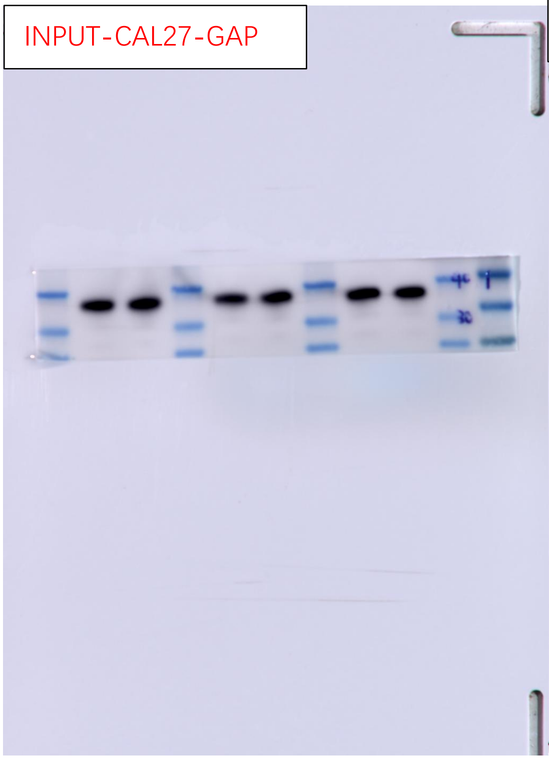

INPUT-CAL27-CHRNA5

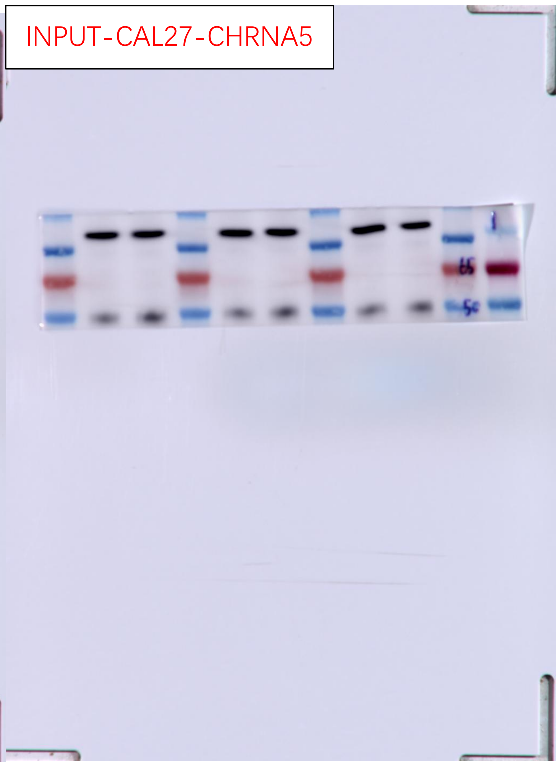

INPUT-CAL27-CES1

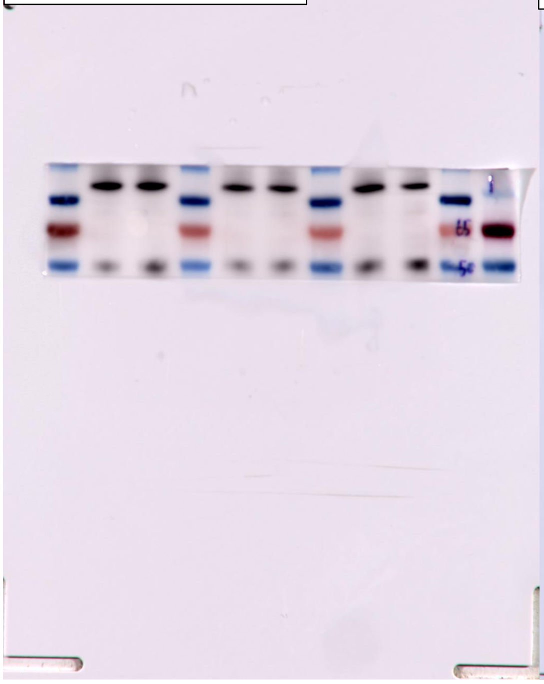

INPUT-CAL27-FULL

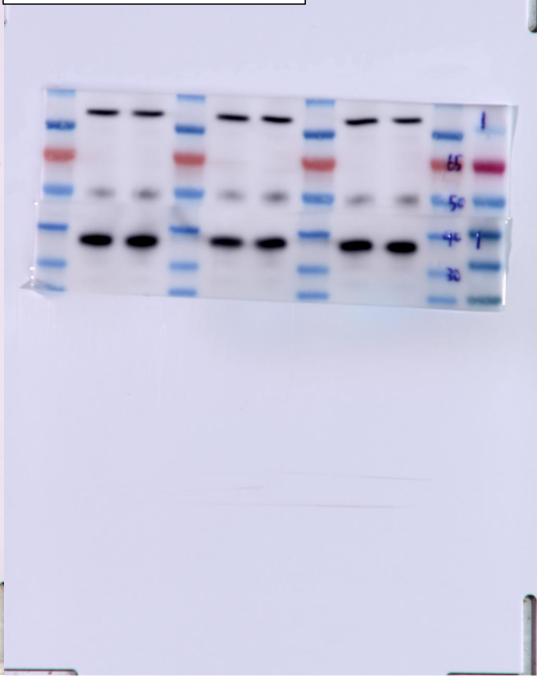

TU686-FLAG

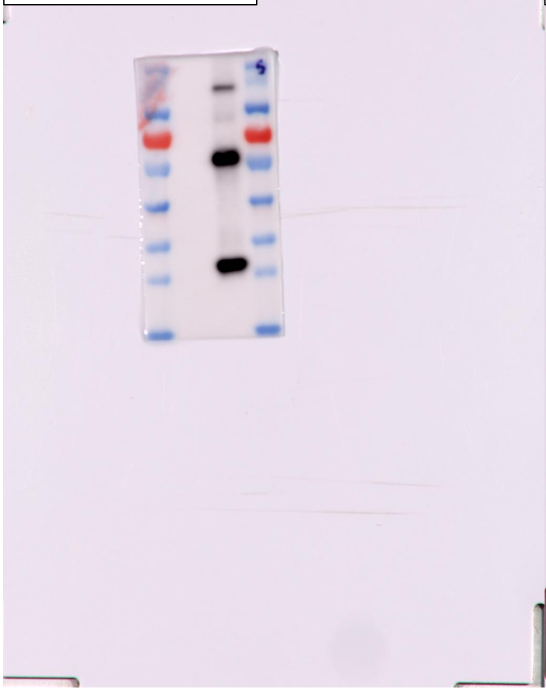

TU686-CES1

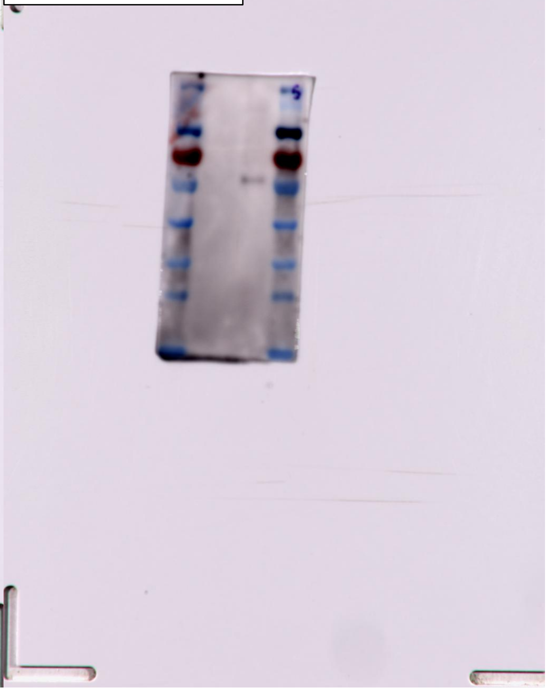

INPUT-TU686-GAP

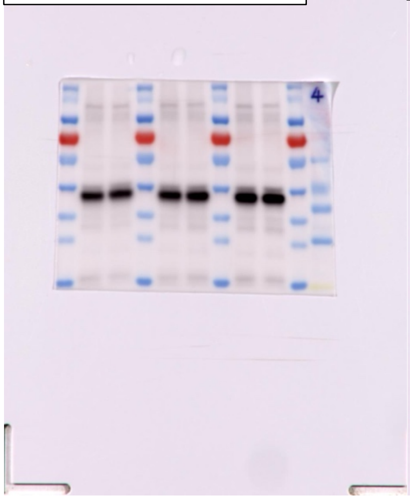

INPUT-TU686-CHRNA5

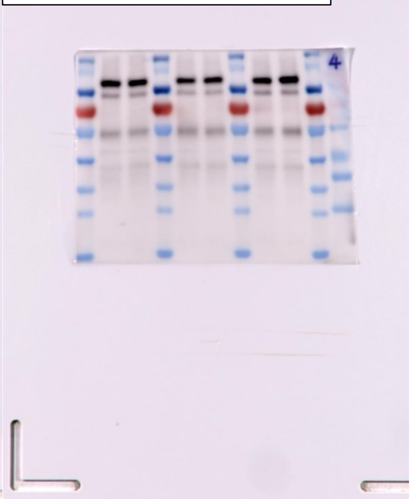

INPUT-TU686-CES1

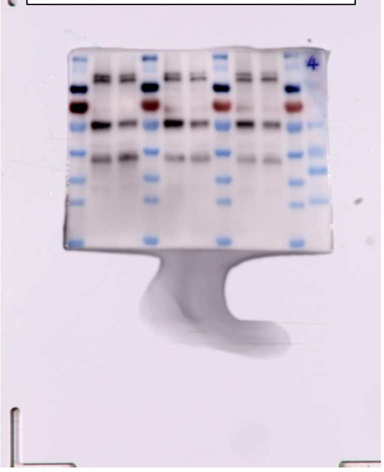

Supplement: Supplementary file 3 — Original Data [file 41419_2024_7178_MOESM3_ESM.zip › Supplemental Material/Figure 7D:E.pdf]
